# Leveraging Conformational and Nitrogen Atom Inversion for Room-Temperature Ferroelectricity

**DOI:** 10.1021/jacs.5c22312

**Published:** 2026-02-23

**Authors:** Alexander Ragins-Da Rosa, Megan Goh, Danny Jeong, Tristan J. Kim, Spencer C. Davis, Ren A. Wiscons

**Affiliations:** † Department of Chemistry, 1180 Amherst College, 25 East Drive, Amherst, Massachusetts 01002, United States; ‡ Department of Physics, Amherst College, 25 East Drive, Amherst, Massachusetts 01002, United States

## Abstract

Nitrogen inversion, in which a pyramidalized tricoordinate nitrogen center turns “inside out”, is an intriguing phenomenon that has inspired a century of fundamental research but has yet to find practical application. In this work, classical nitrogen inversion is used to template polarization switching in a new class of molecular ferroelectrics, material candidates for next-generation digital information storage systems. We demonstrate that azangulene, a bowl-shaped nitrogen-centered heterotriangulene, when adopting a polar crystal packing motif, exhibits above-room-temperature ferroelectricity that we attribute to whole-molecule inversion. Although the mechanism of classical nitrogen inversion predicts a planar transition-state structure, we isolate a crystallographic polymorph in which the bowl depth of azangulene is flat, suggesting that the planar geometry is a stable and isolable structure on the conformational energy surface. A combination of crystallographic polymorphism and computational investigations unravels the complex interplay between the enthalpic and entropic factors contributing to the unique functionality of this molecule.

## Graphical abstract

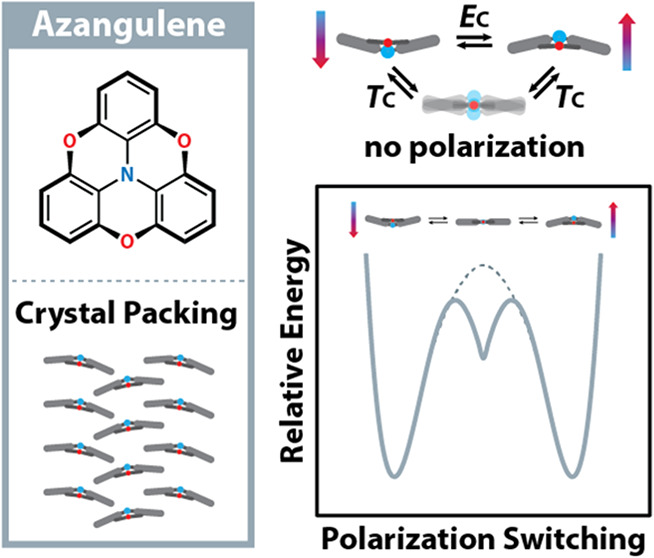

## Introduction

Nitrogen inversion has captured the fascination of researchers across the fields of chemistry and physics since its earliest reported conceptualization in 1924,^1–7^ a topic that recently celebrated its 100-year anniversary. Today, nitrogen inversion is known to take place via two primary mechanisms: by classical traversal of an energetic barrier (Figure 1A) with a planar transition-state structure^4,8–11^ or by quantum tunneling, as is observed in the case of nitrogen inversion in ammonia.^12–15^ Despite a rich foundation in physical chemistry, in practice, nitrogen inversion is typically ignored for achiral nitrogen centers or is intentionally avoided for tricoordinate nitrogen stereocenters, as nitrogen inversion offers a stereoisomerization pathway that can complicate the approval of new chiral pharmaceutical candidates.^16–18^ For this reason, emphasis has been placed on suppressing nitrogen inversion.^16–23^ Despite the hesitation to adopt compounds sensitive to nitrogen inversion for practical applications, in this work, we employ a century of insight into the mechanisms of nitrogen inversion to template polarization switching in the covalent structure of an organic molecular ferroelectric.

**Figure 1:**
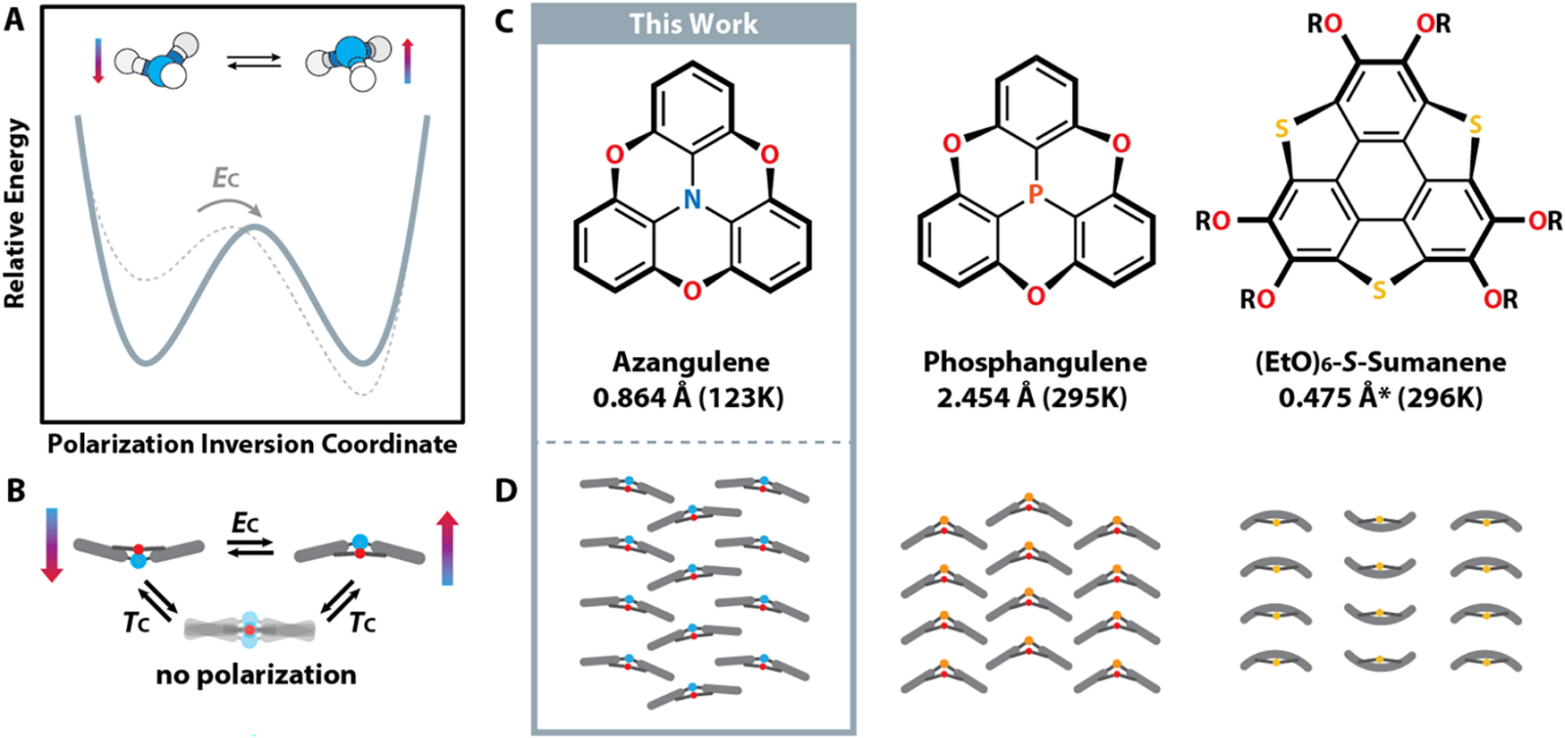
(A) Polarization switching PES for a tricoordinate nitrogen center (solid line) and a generic ferroelectric under a coercive applied *E*-field, *E*_C_ (dashed line); (B) *E*-field-driven azangulene whole-molecule inversion in which *E*_C_ is the coercive electric field, *T*_C_ is the Curie temperature, and blue and red arrows indicate the molecular dipole from electron poor (blue) to electron rich (red); (C) skeletal structures of heteroatom-centered triangulenes and a functionalized *S*-sumanene with their crystallographic bowl depths and the temperatures at which the bowl depths were determined, and are provided below the name of the corresponding molecule; (D) representations of the crystal packing motifs associated with the skeletal structures given in (C). CCDC accession codes: azangulene (262144), phosphangulene (1249452), and (EtO)_6_-*S*-sumanene (1912824). *Bowl depth is the calculated average of the different bowl depths present in the asymmetric unit.

Ferroelectric materials exhibit a remarkable range of electronic properties that emerge from the spontaneous alignment and coerced inversion of electric dipoles (polarization switching).^24–27^ Ferroelectricity has recently garnered broad interest for applications in nonvolatile digital information storage due to the growing demand for high-performance computing resources.^24,26–28^ In 2025, with advancements addressing compatibility challenges between inorganic ferroelectrics and silicon transistors,^28–31^ ferroelectric information storage platforms were estimated to reduce the energy consumption of main memory systems by 21%,^32^ while offering comparable data access speeds relative to the nonferroelectric industry standard.^27^ However, it is challenging to improve the performance of ferroelectric materials through rational chemical modification because changes to the crystal structure often result in the loss of structural instabilities that give rise to ferroelectricity.^33,34^

The mechanism underlying polarization switching in two-state ferroelectrics is governed by a symmetrical double-well potential energy surface (PES), in which two isoenergetic states with opposite electrical polarities are connected by a nonpolar transition state.^24^ Upon application of a coercive electric field, *E*_C_, the symmetry of the double-well PES is broken (Figure 1A), driving the material to preferentially adopt one polarization state that remains kinetically trapped upon removal of the *E*-field. For this reason, coupling between structural instabilities and disfavored polar structural symmetries is inherent to achieving ferroelectricity. In organic molecular ferroelectrics, polarization switching is typically realized through diverse mechanisms^35^ that couple the atomic/molecular order to the lattice symmetry. Crystalline organic ferroelectrics are generally categorized as hosting solid-state cooperative proton transfers (e.g., squaric acid,^36–40^ anthranilic acid,^41^ croconic acid,^42,43^ benzimidazoles,^44^ and phenazine-chloranilic acid^45,46^ ), Peierls distortion-like π-stacking displacements (e.g., tetrathiafulvalene-chloranil, TTF-CA,^38,47,48^ and pyromellitic diimide cocrystals^49^), or whole-molecule disorder (e.g., acenaphthene-2,3,5,6-tetrafluoro-7,7,8,8-tetracyanoquinodimethane, AN-F_4_TCNQ,^50^ and diazabicyclo[2.2.2]­octane, DABCO, salts^34,51,52^ ).

Furthermore, polarization switching in ferroelectrics, particularly organic ferroelectrics, is incompletely understood because mechanistic investigations that require chemical modification of the parent species and/or substitutional replacement cannot be executed without a change in the crystal structure^34^ (e.g., TTF-CA and tetrathiafulvalene-bromanil, TTF-BA^48^), except in rare cases (e.g., substitution of hydrogen with deuterium^35,50,53,54^ ). We present an approach to address this long-standing challenge in the field by embedding the structural and electronic frustration inherent to the mechanism of ferroelectricity into the covalent structure of a small molecule containing a tricoordinate nitrogen center, azangulene, that leverages classical nitrogen atom inversion as the key to producing canonical polarization switching (Figure 1A,B).

It is well accepted that bowl-shaped polycyclic aromatic hydrocarbons (PAHs), such as corannulene derivatives and sumanenes, invert between conformers via a double-well PES, in which the flat geometry is conventionally understood to represent the transition-state structure, except when substituted with sterically demanding groups.^55–57^ The inversion of bowl-shaped molecules, such as sumanene, has been investigated for ferroelectric applications;^58–60^ however, bowl-shaped PAHs do not typically crystallize such that there is a parallel alignment of the molecules in the solid state, resulting in bulk materials that are not electronically polar and in which ferroelectricity is symmetry-disallowed. Bowl-shaped PAHs can be functionalized with aliphatic groups that induce columnar packing motifs.^59,61^ Although ferroelectricity has been demonstrated in these functionalized bowl-shaped PAHs, the aliphatic groups introduce vibrational and rotational modes that are sensitive to the applied electric field,^60,62^ resulting in additional mechanisms of dielectric leakage that reduce the efficiency and thermal stability of the material. In contrast to bowl-shaped PAHs, unsubstituted heteroatom-centered triangulenes show a preference for crystallizing into columnar packing motifs with azangulene and phosphangulene (phosphorus-centered triangulene)^63,64^ also crystallizing into polar bulk materials (Figure 1B,C), making this class of materials an ideal candidate for ferroelectric investigations. Although the origins of polar crystal packing in bowl-shaped heterotriangulenes are complex, investigations by Yamamura et al.^65^ and Heskia et al.^66,67^ have contributed significantly to the understanding of the molecular properties and intermolecular interactions correlated with the crystal packing differences exhibited by this class of compounds.

Here, we report room-temperature ferroelectricity in azangulene single crystals. A combination of X-ray diffraction (XRD), calorimetry, polarization hysteresis, and computational investigations provides converging indirect observations that couple bulk ferroelectricity to whole-molecule (bowl and nitrogen atom) inversion. Azangulene shows rich conformational polymorphism. Although only one of the identified polymorphs fulfills the symmetry requirements for ferroelectricity, we found that the polar azangulene polymorph is the thermodynamically favored solid form at room temperature, ensuring that the ferroelectric phase can be reliably accessed in bulk. For the first time, we found that the previously assumed planar transition-state structure may be a stable and accessible geometry on the conformational energy surface for azangulene. This work not only demonstrates the viability of whole-molecule (bowl and nitrogen atom) inversion as a mechanism to achieve room-temperature ferroelectricity in organic molecular materials but also sheds new light on the thermodynamic drivers of nitrogen atom inversion in the solid state, offering actionable strategies to improve the performance of azangulene-based ferroelectrics.

## Results and Discussion

### Azangulene Inversion toward Polarization Switching

Azangulene, first synthesized by Kuratsu et al.,^68^ can be synthesized in five steps (synthetic details provided in the Supporting Information, Sections S1.1–S1.6). Upon purification, the final product is obtained as a pale yellow crystalline solid, and the structure of azangulene was confirmed using single-crystal X-ray diffraction (SCXRD). Although the previously reported azangulene crystal structure (CCDC 262144) was solved and refined in the *P*2_1_ space group (Figures 2A and S10), we found that azangulene solves in the polar *Cmc*2_1_ space group (Figures 2B and S10 and Table S1). It should be noted that, despite the difference in the selected space groups, the crystal packing is identical between the two structures, and there is good agreement between the reported azangulene geometry and the geometry solved and refined in *Cmc*2_1_. Importantly, the dipole moments of the azangulene molecules are nearly aligned with the polar *c*-axis of the crystal (Figure 2B), indicating that whole-molecule inversion, if energetically accessible, is coupled to bulk polarization switching.

**Figure 2:**
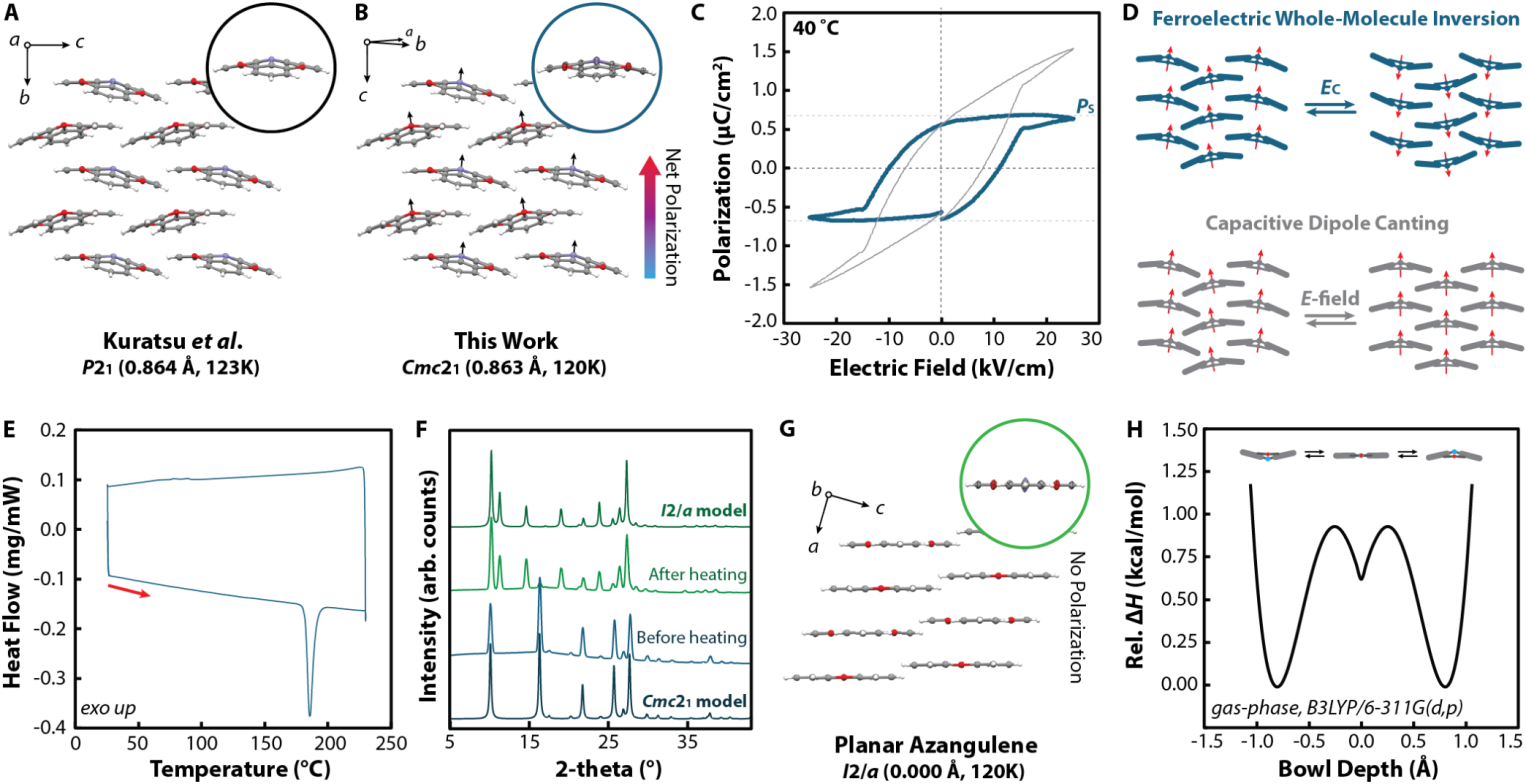
(A) Crystal packing motif solved in *P*2_1_ and single-molecule conformation of azangulene previously published by Kuratsu et al.^68^ (CCDC 262144); (B) crystal packing motif of azangulene solved in *Cmc*2_1_ and single-molecule conformation collected at 120 K; (C) polarization hysteresis loop collected on a single-crystalline device of azangulene at 40 °C. The net polarization loop (gray) was corrected for the linear dielectric (capacitive) component to determine the saturation polarization (*P*_S_). The adjusted loop is shown in blue; (D) molecular representations of the ferroelectric contribution to the net polarization hysteresis loop through whole-molecule inversion (top, blue) and the linear dielectric (capacitive) contributor through whole-molecule canting (bottom, gray); (E) differential scanning calorimetry (DSC) trace of azangulene solved in *Cmc*2_1_, showing an irreversible endotherm at 185 °C; (F) PXRD patterns of azangulene solved in *Cmc*2_1_ (Form I) before and after heating to 200 °C. PXRD patterns calculated (“space group model”) from the SCXRD structures of bowl azangulene (*Cmc*21, Form I) and planar azangulene (*I*2/*a*, Form II) are given in bold at the top and bottom of the plot; (G) crystal packing motif of planar azangulene following the SCSC transformation (Form II, *I*2/*a*) and single-molecule conformation of planar azangulene collected at 120 K; (H) inversion enthalpy surface for a single molecule of azangulene in the gas phase.

Polarization hysteresis (*P-E*) loops measured from single-crystalline devices of azangulene collected at 40 °C (see Sections S1.14 and S1.15) confirm the room-temperature ferroelectricity. The *P*–*E* hysteresis loops show two components (Figure 2C): a ferroelectric component indicated by features of polarization switching and saturation, and a linear dielectric (capacitive) component that tilts the polarization hysteresis loop. We attribute the ferroelectric component to the whole-molecule inversion (Figure 2D, top) and propose that the canting of the azangulene molecules contributes to the capacitive component (Figure 2D, bottom). Given that the linear dielectric component can complicate the determination of ferroelectric parameters, the *P*–*E* loops were corrected for the effects of this component using the Preisach model.^69,70^ From the *P*–*E* loop of the ferroelectric component, the *E*_C_, remnant polarization (*P*_r_), and saturation polarization (*P*_S_) were found to be ±11.0 kV cm^–1^, ±0.60 μC cm^–2^, and ±0.66 μC cm^–2^, respectively. Although modest, these values are in good agreement with the polarization magnitude predicted for azangulene at 40 °C, which was calculated to be 0.81 μC cm^–2^ with the possibility of whole-molecule canting to increase the measured polarization up to 0.92 μC cm^–2^ (see Section S1.17). Additionally, these values are on par with those of ferroelectric sumanenes that undergo bowl-to-bowl inversion, typically achieving *P*_r_ values of 0.4–0.7 μC cm^–2^.^60^ It should be noted that in the absence of the Preisach model correction, *P*_S_ cannot be determined for this system because the raw experimental polarization hysteresis loop does not saturate. Furthermore, the raw *P*_r_ is identical to the Preisach-corrected *P*_r_ because there is a negligible linear dielectric contribution at 0-field. The use of the Preisach model limits the overestimation of *P*_S_ in chemical systems with significant linear dielectric contributions.

A differential scanning calorimetry (DSC) trace was collected for azangulene single crystals (Figure 2E) to determine the Curie temperature, *T*_C_ (see Section S1.13). *T*_C_ delineates the highest temperature at which ferroelectricity is expected because, above this temperature, thermal fluctuations are sufficient to traverse the energy barrier separating the polarization states. The DSC trace reveals an endotherm at 185 °C upon heating without a corresponding exotherm upon cooling, signaling that the material undergoes a thermally irreversible phase transition. XRD measurements performed on the material following the phase transition revealed a single-crystal-to-single-crystal (SCSC) transformation (Figure 2F), after which the complete planarity of the azangulene bowl depth was observed (Figure 2G). This finding suggests that the presumed flat transition-state structure is instead a minimum energy position in the conformational energy landscape for azangulene. Motivated by this finding, the inversion enthalpy surface for a single molecule of azangulene in the gas phase was calculated using density functional theory (DFT) (see Sections S1.16 and S8). The calculated inversion enthalpy surface for azangulene is a triple-well potential (Figure 2H), rather than the canonical double-well potential for classical nitrogen inversion (Figure 1A), successfully predicting that conformationally planar azangulene should be experimentally observable.

### Azangulene Conformational Polymorphism

Azangulene is conformationally polymorphic, demonstrating a change in crystal packing associated with a change in the molecular conformation. Conformational polymorphism offers the rare opportunity to infer the energy relationship of conformers by establishing the thermodynamic relationship between polymorphs.^71,72^ The DSC trace of the ferroelectric polymorph Form I shows that Form I thermally converts to Form II, the polymorph in which azangulene has a planar bowl depth at 185 °C. This SCSC transformation indicates that Form II is the thermodynamic polymorph above 185 °C. We establish that Form I is the room-temperature thermodynamic polymorph by separately subjecting powders of Forms I and II to reversible crystallization conditions by slurrying the material at supersaturation at room temperature for 2 weeks (see Section S1.8). Using powder X-ray diffraction (PXRD), we observe that Form I remains Form I after 2 weeks of equilibration and that Form II converts into Form I (Figure 3A). Given this, we propose that the stabilities of Forms I and II cross above the room temperature (Figure 3B) due to the competition between the enthalpic stability of Form I and the greater entropy change associated with conformational planarization in Form II.

**Figure 3:**
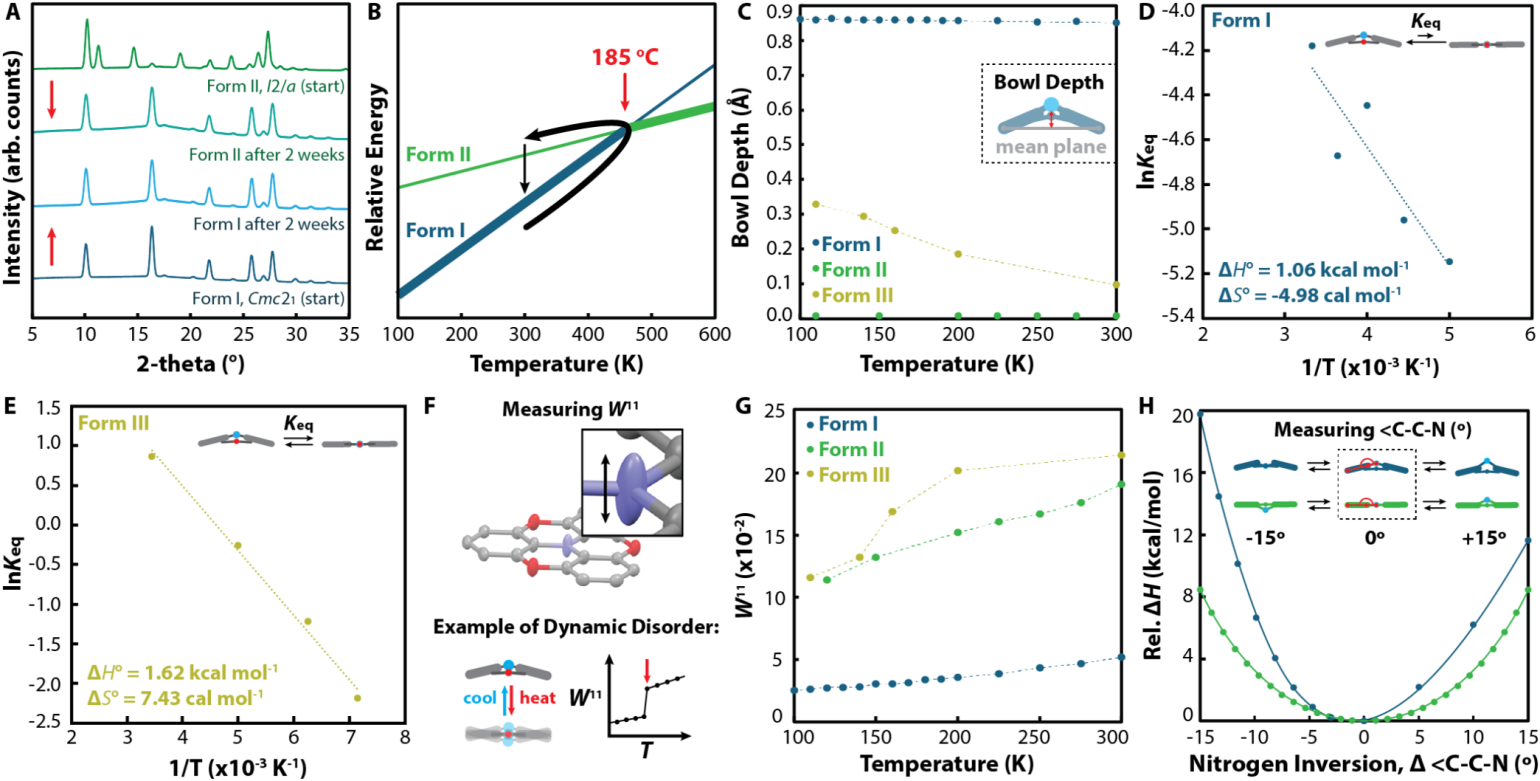
(A) Powder X-ray diffraction (PXRD) patterns before and after phase-pure Form I and phase-pure Form II powders were slurried in benzene for 2 weeks; (B) proposed map of polymorph stabilities constructed from a combination of the discussed DSC and PXRD data, highlighting the crossing of the relative stabilities of Form I and Form II at the phase transition temperature of 185 °C. The bold black arrow shows the solid-state phase behavior of azangulene upon heating above the phase transition and cooling, while the narrow black arrow indicates the phase behavior upon slurry recrystallization at room temperature; (C) experimentally determined bowl depths for a single molecule of azangulene in Form I (blue), Form II (green), and Form III (gold); van’t Hoff plots showing the change in enthalpy and entropy upon planarization of azangulene in (D) Form I and (E) Form III; (F) geometric definition for anisotropic displacement parameter (*W*_N_^11^) measurements; (G) experimentally determined anisotropic displacement parameters for the central nitrogen atom of azangulene parallel to the principal axis of the molecule (*W* ^11^) in Form I (blue), Form II (green), and Form III (gold); (H) energy profiles for nitrogen atom inversion in azangulene adopting a bowl (blue) and planar (green) conformation, in which nitrogen inversion is measured by the difference in the C–C–N angle (indicated in the inset) from 180° (“Δ<C–C–N”).

Although the enthalpy and entropy contributions to the total energy of molecular crystals arise due to a combination of complex intra- and intermolecular factors, we sought to approximate the enthalpy contributions associated exclusively with the molecular conformation of azangulene. Critical to this analysis was the discovery of a third conformational polymorph of azangulene, Form III, in which azangulene adopts a molecular conformation intermediate to those of Form I (bowl) and Form II (planar). The variable-temperature SCXRD structures of all three polymorphs were collected at intervals between 100 – 300 K, and the conformational bowl depth of azangulene present in each structure was measured (Figure 3C, inset and Table S2). The bowl depth is defined as the distance between the central nitrogen atom and the mean plane drawn between the three aryl carbon atoms *para* to the central nitrogen atom.

We observe subtle planarization of azangulene upon heating Form I and significant planarization upon heating Form III (Figure 3C). No changes in the planar bowl depth of azangulene in Form II were observed. From these data, a two-state model was applied to determine the bowl planarization *K*_eq_ values at each temperature (see Sections S1.11 and S4), which were used to construct van’t Hoff plots. The linear regions of the plots were fitted to extract the enthalpy and entropy changes associated with bowl planarization in Forms I (Figure 3D) and III (Figure 3E). For Form I, both Δ*H°* and Δ*S°* were found to be unfavorable for planarization, resulting in a Δ*G*° of +0.4 kcal mol^–1^ at room temperature. In contrast, the planarization of azangulene in Form III is favorable at room temperature (Δ*G°* of −3.8 kcal mol^–1^) due to the favorable entropy change upon planarization. From this analysis, we suggest that the enthalpic stability of Form I (compared to that of Form II) is related to the increased enthalpic stability of the bowl conformer in Form I relative to the planar bowl depth measured in Form II.

To investigate the entropic contributions differentiating Forms I and II, we compared the temperature dependence of the central nitrogen atom’s anisotropic displacement parameter (ADP, *W*_N_^11^) extracted from crystal structures measured between 100–300 K (Figure 3F, Section S1.12, and Table S4). ADPs capture the nonspherical shape of electron density associated with atoms refined from XRD measurements due to a combination of dynamic (vibrational) and static disorder.^73,74^ Although the ADPs associated with a single SCXRD structure cannot unambiguously describe structural dynamics, the temperature evolution of ADPs can be inferred as correlated vibrational motion and/or static disorder (Figure 3F).^74^

In Form I, there is a slight increase in *W*_N_^11^ upon heating from 100 K to room temperature (Figure 3G); however, the change in *W*_N_^11^ is nearly identical to that of the adjacent carbon atom (*W*_C_^11^), indicating that the change in *W*_N_^11^ is attributable to a general increase in thermal fluctuations associated with the increase in data collection temperature and is not related to a change in the geometry or vibrational state of the central nitrogen atom specifically. In Forms II and III, *W*_N_^11^ is significantly greater than the adjacent *W*_C_^11^ at all measured temperatures, indicating that the temperature-dependent change in ADP is specific to the nitrogen center. Forms II and III show a severe elongation of the central nitrogen atom *W*_11_ at all temperatures relative to that of Form I (Figure 3G). For cases in which ADP elongation represents averaged static and positional disorder, it is appropriate to refine the structural model by splitting the occupancy of the elongated atom over multiple positions. For Forms II and III, the SCXRD data do not support splitting the occupancy of the central nitrogen atom due to the worsening of the corresponding refinement statistics. Modeling the central nitrogen atom as localized on a single crystallographic position with an elongated ADP for Forms II and III leads to an improvement of the refinement statistics, which strongly suggests but does not alone conclusively determine the origin of the ADP elongation as dynamic nitrogen inversion. Additional investigations are ongoing to definitively determine the physical origin of the central nitrogen atom disorder (static versus dynamic) in Forms II and III.

The temperature dependence of Form III *W*_N_^11^ values adopts a sigmoidal shape (Figure 3G), showing a step at 140 K and saturation between 200 and 300 K. Combined with the temperature evolution of the Form III bowl depths (0.328 Å at 110 K to 0.097 Å at 300 K), these data suggest that nitrogen inversion is vibrationally “unlocked” upon planarization. This finding was further explored by computing the gas-phase energy profiles for nitrogen atom inversion in the bowl and planar conformers by calculating the energy upon oscillating the nitrogen atom position above and below the crystallographic nitrogen atom position for both conformers while keeping the positions of all other atoms static (see Sections S1.16 and S9). The energy profile for nitrogen inversion in the planar azangulene conformer is significantly flatter/wider than that for nitrogen inversion in the bowl conformer (Figure 3H), suggesting that the energy spacing between the corresponding vibrational states is narrower in the planar azangulene conformer than that in the bowl conformer.

The difference in the thermal evolution of the *W*_N_^11^ values for the three polymorphs points to dynamic nitrogen atom inversion as a possible entropic driving force for the interconversion between Forms I and II and planarization of the azangulene bowl depth. This was further investigated by quantifying the thermal responsiveness of the polymorphs along the nitrogen inversion axes by measuring the coefficients of the linear thermal expansion (CLTE). The CLTE values were determined along the molecular inversion axes for Form I (*c*-axis) and Form II (*a*-axis) from SCXRD structures measured between 300–400 K (Table S3). The CLTE for Form II is 181.7 MK^–1^, more than triple the CLTE for Form I (58.7 MK^–1^), indicating that the nitrogen inversion axis of Form II is significantly more responsive to changes in temperature than that of Form I, supporting the hypothesis that dynamic nitrogen inversion in the planar conformer is a significant thermodynamic driving force for conformational planarization and the SCSC transformation.

### Physical Origin of Azangulene Conformational Triple-Well Potential

The computed triple-well inversion enthalpy surface for azangulene indicates that the planar conformer is enthalpically stable relative to the transition state connecting the bowl and planar geometries. To better understand the enthalpic stability of azangulene’s planar geometry, the DFT results of azangulene’s conformational enthalpy surface for the whole-molecule inversion were analyzed in greater detail. We found that two competing parameters produced the features of the triple-well potential: (*i*) distortion of the bridging oxygen atom geometry and (*ii*) delocalization of the nitrogen atom’s nonbonding electron pair (Figure 4A and Section S8). The aryl-oxygen bond length and the angles between the bonds formed to the oxygen atom are compressed in the transition-state structure that separates the bowl and planar states. Separately, the partial charge on the central nitrogen atom is neutralized upon planarization due to the improved orbital overlap in the planar geometry relative to the bowl geometry. This effect is visualized using nucleus-independent chemical shift (NICS)^75^ ring current maps (Section S10). The combination of geometric strain, which is minimized in the bowl conformer (Figure 4B), and stabilizing electronic effects, which are maximized in the planar conformer (Figure 4C), results in the computed triple-well potential.

**Figure 4:**
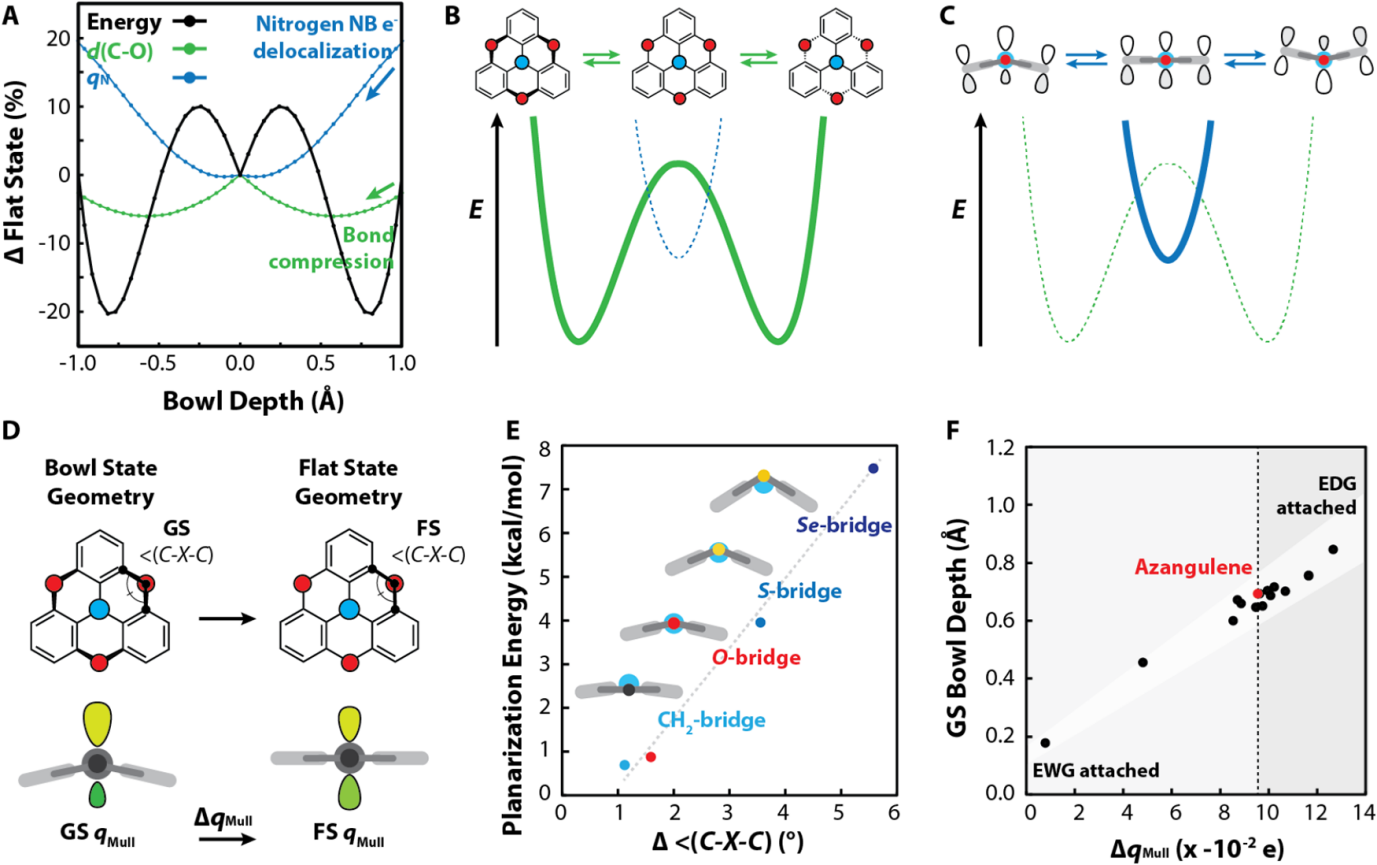
(A) Plot showing the relationship between the computed energy, aryl-oxygen bond length, *d*(C–O), and partial charge on the central nitrogen atom, *q*_N_, during the whole-molecule inversion; sketch of the two major contributors to the triple-well potential energy surface: (B) geometric strain of the bridging atom and (C) stabilization of the nonbonding electron pair on the central nitrogen atom; (D) profile and top-down views of azangulene in the bowl state (GS) and flat state (FS), highlighting the definitions of the bridging angles, <(C–X–C), and nitrogen atom partial charges (Mulliken populations), *q*_Mull_; (E) change in the bridging atom angle between the GS and FS, Δ<(C–X–C), plotted against the planarization energy; (F) change in partial charge of the central *N* atom (Δ*q*_Mull_) plotted against the GS bowl depth.

The roles of the bridging atom, geometric strain, and electronic effects were further explored by constructing a computational library of azangulene derivatives (Sections S1.16). The full library of compounds and their relevant computed properties is listed in Section S11. For each compound in the computational library, the molecular symmetry was restrained to the *C*_3*v*_ point group, and the geometry was optimized to compute molecular properties at the equilibrium bowl depth. This was repeated for the compound constrained to the *D*_3*h*_ point group to compute the properties of the planar geometry. The outputs for this pair of calculations were compared for each compound in the library. Notably, we found that larger bridging atoms that form longer average σ-bonds to aryl groups yield deeper equilibrium bowl depths and a greater energy difference between the bowl and flat conformers (Figure 4D,E), a trend that is contrary to the reported bowl depth trends in sumananes^55,60^ and phosphangulenes.^65^ We also found that electron-withdrawing groups flatten the equilibrium bowl depth, while electron-donating groups deepen the equilibrium bowl depth (Figure 4D,F), a trend that reveals the role of aryl functionalization in modulating the delocalization of the central nitrogen atom’s nonbonding electron pair. This finding is consistent with previous reports^76^ describing the computed relationship between the electronic effects and aniline nitrogen atom pyramidalization/planarization.

From these computational investigations, we propose that the experimental observation of the planar azangulene geometry arises primarily due to two factors: (*i*) enthalpic stabilization of the central nitrogen atom’s nonbonding electron pair through resonance delocalization and stabilizing inductive electronic effects introduced by the bridging oxygen atoms, and (*ii*) entropic stabilization induced by dynamic nitrogen inversion, which is observed in the flat geometry but not observed in the bowl conformer. Given this analysis, we propose that functionalizing azangulene with electron-donating groups could minimize the possible kinetic trapping effects of the planar geometry on ferroelectric polarization switching.

## Conclusions

The mechanistic similarities between classical nitrogen inversion and polarization switching led us to target azangulene as a promising ferroelectric candidate. A combination of polarization hysteresis measurements performed on single crystals of Form I azangulene, XRD investigations, calorimetry, and computational studies provides converging indirect support, suggesting that room-temperature ferroelectricity can be facilitated, at least in part, by nitrogen atom inversion. Critically, we introduce a mechanistically-forward approach to designing and targeting novel ferroelectric materials with the goal of producing a class of organic ferroelectrics amenable to thorough fundamental investigation and reliable performance engineering strategies.

## Supplementary Material



## Notes

Experimental details, X-ray diffraction data, single-crystal X-ray structures, PXRD patterns, DSC traces, ferroelectric testing data, and DFT calculations. (PDF)
