# Supplementary material for: Leveraging Conformational and Nitrogen Atom Inversion for Room-Temperature Ferroelectricity
Source: J Am Chem Soc. 2026 Feb 23;148(9):9901–9. doi: 10.1021/jacs.5c22312 (PMC12983323; doi:10.1021/jacs.5c22312)
Supplement: Supplementary file 1 [file ja5c22312_si_001.pdf]

Supporting Information

Leveraging conformational and nitrogen atom inversion for room-temperature ferroelectricity

*Alexander Ragins-Da Rosa[1], Megan Goh[2], Danny Jeong[1], Tristan J. Kim[1], Spencer C. Davis[1,2], and Ren A. Wiscons[1]**
[1]Department of Chemistry, Amherst College, 25 East Dr. Amherst, Massachusetts, 01002 USA
[2]Department of Physics, Amherst College, 25 East Dr. Amherst, Massachusetts, 01002 USA

**E-mail: rwiscons@amherst.edu*

Table of Contents:

SI1. Experimental Methods
*SI1.1. Synthesis of 2-bromo-1-(3-fluoro-2-nitrophenoxy)-3-methoxybenzene (**1**)*
*SI1.2. Synthesis of 2-bromo-1-(3-(2-bromo-3-fluorophenoxy)-2-nitrophenoxy)-3-methoxybenzene (**2**)*
*SI1.3. Synthesis of 2-(2-bromo-3-fluorophenoxy)-6-(2-bromo-3-methoxyphenoxy)aniline (**3**)*
*SI1.4. Synthesis of 1-fluoro-13-methoxybenzo[1,4]oxazinophenoxazine (**4**)*
*SI1.5. Synthesis of 1-fluoro-13-benzo[1,4]oxazinophenoxazinol (**5**)*
SI1.6. Synthesis of azangulene
SI1.7. Azangulene Recrystallization Protocols
SI1.8. Slurry Crystallization Protocol
SI1.9. Single-Crystal X-ray Diffraction Methods
SI1.10. Powder X-ray Diffraction Methods
SI1.11 Method for Determining K_{eq} from Crystallographic Bowl Depths
SI1.12. Method for Extracting Anisotropic Displacement Parameters
SI1.13. Differential Scanning Calorimetry
SI1.14. Device Preparation for Azangulene Ferroelectric Devices
SI1.15. Ferroelectric Testing Measurements
SI1.16. Computational Methods
SI1.17. Prediction of Polarization Magnitude for Form I Azangulene
SI2. Table of Crystallographic Parameters
SI3. Variable-Temperature Crystallographic Parameters for Azangulene Solid Forms
SI4. van't Hoff Analysis of Form I and Form III Crystallographic Bowl Depths
SI5. Polarization Hysteresis Measurement
SI6. Differential Scanning Calorimetry
SI7. Slurry Equilibration of Azangulene Solid Forms
SI8. Computational Investigation of Gas-Phase Azangulene Whole-Molecule Inversion
SI9. Computational Investigation of Gas-Phase Azangulene Nitrogen Atom Inversion Enthalpy Surface
SI10. Nucleus-Independent Chemical Shift (NICS) Ring Current Maps
SI11. Computational Library of Azangulene Derivatives
SI12. ORTEP for Determined X-Ray Crystal Structures
SI13. References

SI1. Experimental Methods

All reagents were used as received from suppliers without additional purification. Sodium hydride (NaH, 60% (w/w) dispersion of NaH in mineral oil), 2-bromo-3-methoxyphenol, 5% (w/w) palladium on carbon, hydrazine hydrate (80% solution in water), JohnPhos (97%), *tris*(dibenzylideneacetone)dipalladium(0) ($Pd^0_2(dba)_3$), sodium *tert*-butoxide (NaO^tBu), boron tribromide (BBr_3, 2M in DCM), anhydrous potassium carbonate (K_2CO_3), anhydrous magnesium sulfate ($MgSO_4$), anhydrous dimethyl sulfoxide (DMSO, >99.9%), ethanol (200 proof), anhydrous methanol, toluene (>99.5%), dichloromethane (DCM), dimethylformamide (DMF), and benzene were obtained from Sigma Aldrich. Hexanes, ethyl acetate, and acetone were obtained from Alfa Aesar. 2,6-difluoronitrobenzene and 2-bromo-3-fluorophenol were purchased from TCI. Toluene and DMF were dried over 3 Å molecular sieves for at least one day prior to use.

*SI1.1. Synthesis of 2-bromo-1-(3-fluoro-2-nitrophenoxy)-3-methoxybenzene (**1**)*

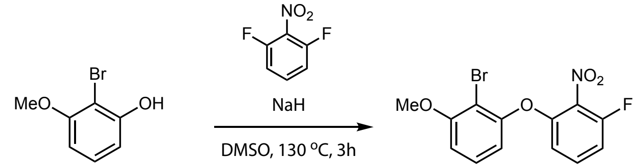

2-bromo-3-methoxyphenol   Intermediate ***1***

Scheme SI1. Summary of reaction conditions to convert 2-bromo-3-methoxyphenol into intermediate ***1***.

The synthesis of azangulene was adapted from previously reported literature procedure.[1] A 60% (w/w) dispersion of NaH in mineral oil (108 mg, 2.700 mmol) and DMSO (7 mL) were mixed in a sealed three-neck round-bottom flask under nitrogen atmosphere at 60 °C for 1 h. A solution of 2-bromo-3-methoxyphenol (500 mg, 2.463 mmol) in DMSO (2 mL) was added to the reaction mixture and stirred at 60 °C for 1 h. The flask was cooled to room temperature, and a solution of 2,6-difluoronitrobenzene (396 mg, 2.490 mmol) in anhydrous DMSO (1 mL) was added to the reaction mixture. The flask was heated to 130 °C and stirred for 3 h. The reaction solution was cooled to room temperature and quenched with water, precipitating the crude product. The crude product was extracted into ethyl acetate (2 x 30 mL) and the combined organic fractions were washed with water (2 x 30 mL), brine (2 x 30 mL), and dried over anhydrous $MgSO_4$. The material was concentrated under reduced pressure to yield intermediate ***1*** as a white powder (769 mg, 2.248 mmol, 91.3% yield). ^1H NMR spectrum of the purified powder is consistent with previously reported literature.[1]

*SI1.2. Synthesis of 2-bromo-1-(3-(2-bromo-3-fluorophenoxy)-2-nitrophenoxy)-3-methoxybenzene (**2**)*

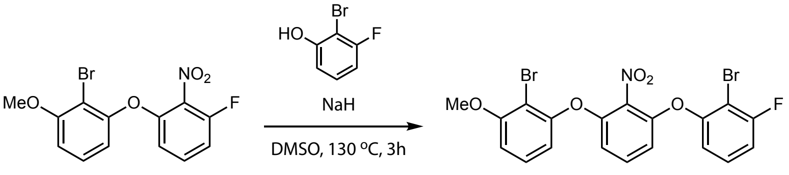

Intermediate ***1***   Intermediate ***2***

Scheme SI2. Summary of reaction conditions to convert intermediate ***1*** into intermediate ***2***.

A 60% (w/w) dispersion of NaH in mineral oil (99 mg, 2.475 mmol) and anhydrous DMSO (6 mL) were mixed in a sealed three-neck round-bottom flask under nitrogen atmosphere at 60 °C for 1 h. A solution of 2-bromo-3-fluorophenol (430 mg, 2.251 mmol) in DMSO (1 mL) was added to the reaction mixture and stirred at 60 °C for 1 h. The flask was cooled to room temperature, and a solution of intermediate ***1*** (769 mg, 2.248 mmol) in anhydrous DMSO (3 mL) was added to the reaction mixture. The flask was heated to 130 °C and stirred for 4 h. The reaction solution was cooled to room temperature and quenched with water, precipitating the crude product. The crude product was extracted into ethyl acetate (2 x 30 mL) and the combined organic fractions were washed with water (2 x 30 mL), brine (2 x 30 mL), and dried over anhydrous $MgSO_4$. The material was concentrated under reduced pressure and washed with ethanol to yield intermediate ***2*** as an off-white powder (730 mg, 1.423 mmol, 63.3% yield). ^{1}H NMR spectrum of the purified powder is consistent with previously reported literature.[1]

*SI1.3. Synthesis of 2-(2-bromo-3-fluorophenoxy)-6-(2-bromo-3-methoxyphenoxy)aniline (**3**)*

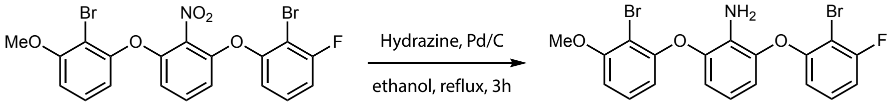

Intermediate ***2*** Intermediate ***3***

Scheme SI3. Summary of reaction conditions to convert intermediate ***2*** into intermediate ***3***.

The synthesis of intermediate ***3*** was adapted from previously reported literature procedure.[1,2] Intermediate ***2*** (730 mg, 1.423 mmol) was mixed in a 100 mL three-neck round-bottom flask with 5% (w/w) palladium on carbon (91 mg), and 80% hydrazine hydrate (1.73 mL) in ethanol (25 mL) under nitrogen atmosphere. The reaction mixture was heated to 80 °C, and stirred for 3 h. The reaction was filtered through a celite pad, and the material was concentrated under reduced pressure. The crude product was recrystallized from methanol to yield intermediate ***3*** as a colorless powder (437 mg, 0.905 mmol, 63.6%). ^{1}H NMR spectrum of the purified powder is consistent with previously reported literature.[1]

*SI1.4. Synthesis of 1-fluoro-13-methoxybenzo[1,4]oxazinophenoxazine (**4**)*

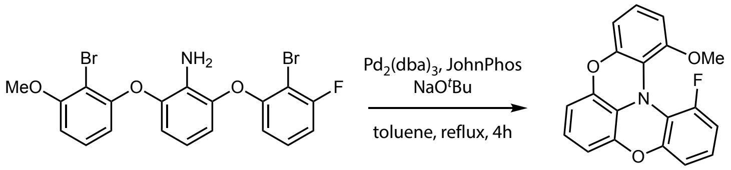

Intermediate ***3*** Intermediate ***4***

Scheme SI4. Summary of reaction conditions to convert intermediate ***3*** into intermediate ***4***.

The synthesis of intermediate ***4*** was adapted from previously reported literature procedure.[1,2] A mixture of $Pd_2(dba)_3$ (49 mg, 0.053 mmol) and JohnPhos (23 mg, 0.078 mmol) in dry toluene (10 mL) was sparged with nitrogen gas in a 100 mL three-neck round-bottom flask for 15 minutes. A suspension of intermediate ***3*** (500 mg, 1.035 mmol) in dry toluene (5 mL) sparged with nitrogen gas was added to the reaction flask with the catalyst. NaO^{t}Bu (298 mg, 3.103 mmol) was subsequently added to the reaction. The flask was refluxed and stirred for 4 h. Following reflux, the mixture was cooled to room temperature and filtered

through a celite pad. The filtrate was diluted with ethyl acetate and washed with 1 M NaOH (2 x 30 mL) and water (2 x 30 mL) and dried over anhydrous $MgSO_4$. The material was concentrated under reduced pressure to a black oil. The black oil was purified by column chromatography on silica gel using 30% (v/v) ethyl acetate in hexanes as the mobile phase. Column fractions were combined to yield intermediate ***4*** as a colorless powder (179 mg, 0.557 mmol, 53.8% yield). ^{1}H NMR spectrum of the purified powder is consistent with previously reported literature.[1]

*SI1.5. Synthesis of 1-fluoro-13-benzo[1,4]oxazinophenoxazinol (**5**)*

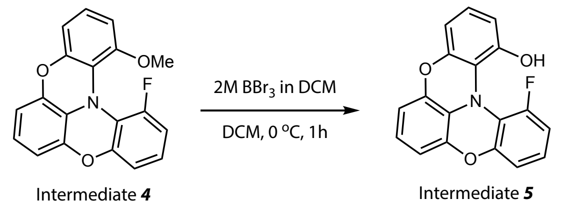

Scheme SI5. Summary of reaction conditions to convert intermediate ***4*** into intermediate ***5***.

The synthesis of intermediate ***5*** was adapted from previously reported literature procedure.[1,2] Intermediate ***4*** (100 mg, 0.311 mmol) was dissolved in DCM (10 mL) in a sealed 100 mL round bottom flask under nitrogen atmosphere. The solution was cooled to 0 °C and BBr_3 (1.4 mmol, 0.7 mL of 2M solution in DCM) was added dropwise to the solution of ***4***. After the addition of BBr_3, the reaction solution was warmed to room temperature and stirred for 1h. The reaction solution was quenched with methanol, dried over anhydrous $MgSO_4$, and concentrated under reduced pressure to an oil. The oil was purified by column chromatography on silica gel using DCM as the mobile phase. Column fractions were combined and concentrated to yield intermediate ***5*** as a colorless powder (92 mg, 0.298 mmol, 96% yield).

SI1.6. Synthesis of azangulene

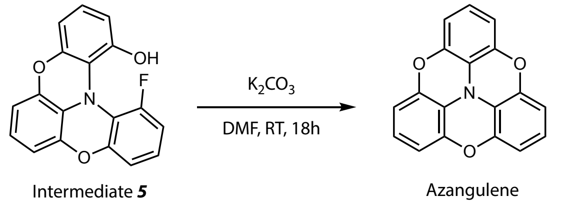

Scheme SI6. Summary of reaction conditions to convert intermediate ***5*** into azangulene.

Intermediate ***5*** (92 mg, 0.298 mmol) was dissolved in dry DMF (2 mL) in a sealed 100 mL round bottom flask under nitrogen atmosphere. A solution of K_2CO_3 (101 mg, 0.731 mmol) in dry DMF (3 mL) was added dropwise to the solution of ***5***. The reaction stirred at room temperature for 18h, after which the reaction was quenched with water and the crude product extracted into ethyl acetate (2 x 30 mL). The combined organic fractions were washed with water (2 x 30 mL) and brine (2 x 30 mL) and dried over anhydrous $MgSO_4$. The solution was concentrated to an oil under reduced pressure and purified by column chromatography on silica gel using benzene as the mobile phase. Column fractions were combined and concentrated to yield azangulene as a pale yellow crystalline solid (49 mg, 0.171 mmol, 57.4% yield). ^{1}H NMR spectrum of the purified powder is consistent with previously reported literature.[1]

SI1.7. Azangulene Recrystallization Protocols

Single crystals of Form I were recrystallized from acetone. Azangulene was added to a 1-dram vial and boiling acetone was slowly added until complete dissolution. The vial was placed in a freezer (-20 °C) overnight, selectively precipitating single crystals of azangulene adopting the Form I crystal packing. Single crystals of Form II were prepared by single-crystal-to-single-crystal (SCSC) transformation of Form I or from the melt phase. The SCSC transformation was executed by heating a sealed 1-dram vial of Form I to 200 °C for 30 min. Alternatively, azangulene melts at 287 °C and the Form II polymorph recrystallizes selectively from the melt phase. Form III was prepared through an evaporative crystallization process at room temperature from a solution of azangulene in acetone and trace DMF.

SI1.8. Slurry Crystallization Protocol

The room-temperature thermodynamic polymorph of azangulene was determined by slurry crystallization at room temperature. Phase-pure Form I and Form II azangulene were loaded into separate 1-dram vials, each with a small Teflon-coated stirbar. The solid was wetted with either acetone or benzene, ensuring that, at equilibrium, not all solid dissolved at room temperature. The vials were capped and slurried for two weeks. The bulk structural phases of azangulene before and after slurrying was characterized by powder X-ray diffraction.

SI1.9. Single-Crystal X-ray Diffraction Methods

X-ray diffraction images were collected using a Rigaku XtaLAB Synergy-i or Rigaku XtaLAB Synergy-S X-ray diffractometer configured in a kappa goniometer geometry. The diffractometer is equipped with a variable-temperature device and a PhotonJet-S microfocus Cu source (λ = 1.54187 Å) and operated at 50 kV and 1 mA. X-ray intensities were measured between 100 K and 400 K with the HyPix detector placed 44.00 mm from the sample. The data were processed with CrysAlisPro version 41_64.117a (Rigaku Oxford Diffraction)[3] and corrected for absorption. The structures were determined in OLEX2[4] using SHELXT[5] and refined using SHELXL.[6] All non-hydrogen atoms were refined anisotropically with hydrogen atoms placed at idealized positions. Single crystals were mounted on a 150 µm MiTeGen MicroMount using mineral oil.

SI1.10. Powder X-ray Diffraction Methods

X-ray diffraction images were collected using a Rigaku XtaLAB Synergy-i X-ray diffractometer configured in a kappa goniometer geometry. The diffractometer is equipped with a variable-temperature device and a PhotonJet-S microfocus Cu source (λ = 1.54187 Å) and operated at 50 kV and 1 mA. X-ray intensities were measured at room temperature with the Bantam detector placed 50.00 mm from the sample. All images were collected with a continuous ϕ-rotation scan with a 300 second exposure. The images were integrated and processed into diffraction patterns using CrysAlisPro version 41_64.117a (Rigaku Oxford Diffraction) [3]. Samples were mounted on a 150 µm MiTeGen MicroMount using Paratone oil. This data collection strategy minimized the effects of preferred orientation.

SI1.11 Method for Determining K_{eq} from Crystallographic Bowl Depths

Conformational bowl depths for azangulene were measured from crystal structures using the Mercury software (Cambridge Crystallographic Data Centre)[7]. A mean plane was calculated between three carbon atoms *para* to the carbon atoms directly

bound to the central nitrogen atom and the distance between the nitrogen atom and the mean plane was taken to be the bowl depth. The equilibrium constant, K_{eq}, for planarization was approximated by assuming that the crystallographic bowl depth is a superposition between: 1) the low-temperature equilibrium bowl conformation specific to each polymorph and 2) the planar conformer. The populations of these two conformers for Forms I and III are given for each temperature in Section SI4. The relative populations of these two conformers were then used to calculate a bowl planarization equilibrium constant, K_{eq}. A similar approach has been used to measure the energetics of nitrogen inversion in the solid state by others,[8] although the relative populations of conformers were determined from a comparison of crystallographic occupancies of disordered parts.

SI1.12. Method for Extracting Anisotropic Displacement Parameters

Anisotropic displacement parameters (ADPs) were treated according to previously reported literature procedure.[9,10] Six values describing the anisotropy were collected from a plain text *CIF file*. These values were matricized according to the order described in the file header. Diagonalization in Mathematica provided three eigenvalues describing the principal axes of an ellipsoid in the XYZ coordinate system. The minor axes – already close in value – were averaged. A ratio between the average and the major axis was taken at each temperature to track the elongation/contraction of electron density across our samples. A value closer to 1 indicates an even distribution of electron density while a value closer to 0 represents an elongation along the major axis.

SI1.13. Differential Scanning Calorimetry

Differential scanning calorimetry (DSC) traces were measured on a Mettler Toledo Instrument DSC 3$^+$ equipped with a Hubber TC100 cooling system under nitrogen atmosphere (50 mL/min). All experiments were run in hermetic aluminum DSC pans with a heating rate of either 5 °C/min or 10 °C/min, covering a temperature range of 25 °C to 295 °C. DSC traces were analyzed using METTLER STARe default data viewer.

SI1.14. Device Preparation for Azangulene Ferroelectric Devices

Devices of Form I azangulene single crystals were prepared by immobilizing crystals on a 1 cm x 1 cm glass slide with minimal vacuum grease. Guided by the crystal face indexation package available in CrysAlis Pro (Rigaku), the immobilized crystal was cleaved in two positions along the long morphological axis of the crystal such that the distance between the cleaved faces was between 10 - 20 μm. Silver contacts (Dupont Micromax™ 4922N) were placed by intercalating silver paint onto the cleaved faces of the immobilized azangulene single crystals using 50 μm diameter aluminum wire. Gold wire (30 μm diameter) was adhered to the silver pads to make a two-point capacitance measurement.

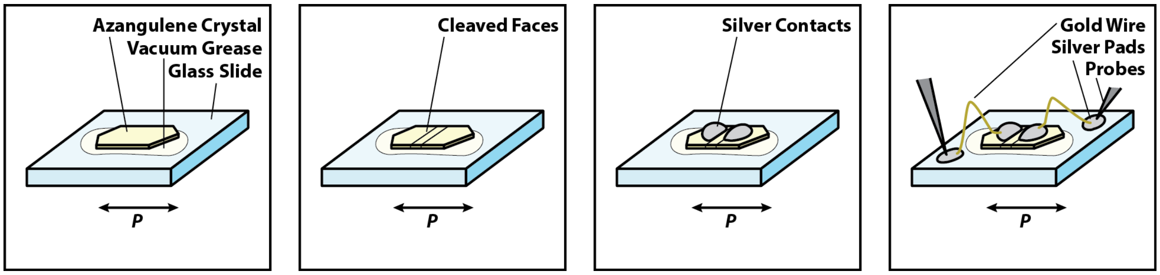

Figure SI1. Schematic of single-crystal device preparation for azangulene ferroelectric measurements.

SI1.15. Ferroelectric Testing Measurements

Polarization hysteresis loops were collected using a Precision Multiferroic II Test System (Radiant Technologies, Inc.) using Vision Software and a variable-temperature four-point probe station (Linkam Scientific). Polarization hysteresis loops were collected between 22 °C and 125 °C and all presented polarization hysteresis loops were collected between +/-75 V and at 500 Hz with a pre-pulse loop. Device dimensions were used to convert the measured capacitance into net polarization and the drive voltage into electric field. Samples were measured in triplicate showing good agreement ($sP_r < 0.1$ μC cm^{-2}) between crystals grown from the same batch. A representative polarization hysteresis loop from a crystal grown from benzene is given in Section SI5.

SI1.16. Computational Methods

Gas-Phase Azangulene Whole-Molecule Inversion: Density functional theory (DFT) was applied to a series of azangulene geometries to determine the molecular energy as the compound undergoes conformational inversion. Using Gaussian software[11] package *via* the WebMO[12] GUI, the bowl conformer and flat conformer geometries extracted from the Form I and Form II crystal structures were geometry optimized. To ensure the flat conformer remained flat through the geometry optimization, the molecular symmetry was restrained to the D_{3h} point group. A Python script, taking two **.xyz files* as input, ensured rotational alignment between the two optimized conformers by applying a rotation matrix to the bowl conformer. A Python script was then used to interpolate fifteen geometries between the two optimized conformers, and similar script was used to extrapolate five additional geometries that simulated a more severe puckering of azangulene's optimized bowl depth. The Gaussian software package via the WebMO GUI was used to determine the energy of each geometry. Energy calculations were repeated for identical series of azangulene geometries using the B3LYP functional with the 6-31G(d) basis set, the B3LYP functional with the 6-311+G(2d,p) basis set, the ωB97XD functional with the def2-TZVP basis set, and the M062X functional with the def2-TZVP basis set. There was general agreement across the results from each method and the presented data were calculated using the B3LYP functional with the 6-311+G(2d,p) basis set.

Gas-Phase Azangulene Nitrogen Atom Inversion: Møller-Plesset (MP) perturbation theory was applied to a series of azangulene geometries to determine the molecular energy as the compound undergoes nitrogen atom inversion. Using the Spartan'20[13] software package, hydrogen atom positions were relaxed for the experimental crystallographic geometries of azangulene in Form I and Form II using density functional theory B3LYP/6-31G(d). Following relaxation of the hydrogen atom positions, all atoms, with the exception of the central nitrogen atom, were 'frozen' to restrain the conformation of the molecular during calculations. A series of geometries representing nitrogen atom inversion were generated for the bowl and planar conformer by oscillating the nitrogen atom position above and below the crystallographic nitrogen atom position. This was executed by constraining the angle between the nitrogen atom and the mean plane of an adjacent ring system. Molecular energies were calculated using MP2 and the 6-311+G(2d,p) basis set for the series of geometries.

Computational Library of Azangulene Derivatives: Density functional theory (DFT) was applied to a series of azangulene derivatives using Gaussian[9] software package *via* the WebMO[10] GUI to determine the role of electronic effects and steric effects on azangulene analogues. For each compound selected for the computational library, the molecular symmetry was restrained to the C_{3v} point group and geometry optimized using the B3LYP functional with the 6-311+G(2d,p) basis set in order to compute

molecular properties at the equilibrium bowl depth and repeated for the compound restrained to the D_{3h} point group to compute properties for the planar geometry. The outputs for this pair of calculations were compared for each compound in the library.

SI1.17. Prediction of Polarization Magnitude for Form I Azangulene

The predicted polarization magnitude was calculated by taking the vector sum of the molecular dipoles computed for a single molecule of azangulene (B3LYP/6-311+G(d,p)) and normalizing this by the unit cell volume measured at 40 °C. The molecular dipole for azangulene is computed at 0.8491 D, although only ~88% of the molecular dipole is aligned along the crystallographically polar axis due to slight canting of individual molecules away from the polar axis. The impact of canting molecules towards the polar axis on the predicted polarization magnitude was computed by assuming 100% of the molecular dipole is aligned along the crystallographically polar axis. Using a *Z* of 4 molecules ($Cmc2_1$ and Z' = 0.5), the dipole of the unit cell was determined in units of μC·cm. This value was normalized by the unit cell volume at 40 °C in units of cm^3 to calculate the polarization in units of $\mu C \cdot cm^{-2}$.

SI2. Table of Crystallographic Parameters

Table SI1. Crystallographic parameters for azangulene Forms I, II, and III.

Compound name	Form I – 120K	Form I – 300K	Form II – 150K	Form III – 160K
CCDC Deposition Number	2513379	2513380	2513381	2513382
Chemical formula	$C_{18}H_9NO_3$	$C_{18}H_9NO_3$	$C_{18}H_9NO_3$	$C_{18}H_9NO_3$
M_r	287.26	287.26	287.26	287.26
Crystal system	*Orthorhombic*	*Orthorhombic*	*Monoclinic*	*Monoclinic*
Space group	$Cmc2_1$	$Cmc2_1$	$I2/a$	$P2_1/n$
Temperature (K)	120	300	150	160
a (Å)	17.4461(3)	17.5297(3)	7.3268(3)	18.585(3)
b (Å)	10.0827(3)	10.1232(3)	10.3596(4)	3.7892(6)
c (Å)	6.82870(10)	6.92640(10)	15.7620(6)	18.943(3)
α (°)	90	90	90	90
β (°)	90	90	94.800(4)	115.37(2)
γ (°)	90	90	90	90
V (Å^3)	1201.19(4)	1229.14(5)	1192.18(8)	1205.3(4)
Z	4	4	4	4
Radiation type	Cu *K*α	Cu *K*α	Cu *K*α	Cu *K*α
μ (mm^{-1})	0.90	0.88	0.91	0.90
Crystal size (mm)	0.20 x 0.14 x 0.03	0.21 x 0.13 x 0.02	0.50 x 0.07 x 0.04	0.13 x 0.05 x 0.03
Data Collection				
Diffractometer	XtaLAB Synergy-S	XtaLAB Synergy-S	XtaLAB Synergy-i	XtaLAB Synergy-i
Absorption correction	Multi-scan	Multi-scan	Multi-scan	Multi-scan
T_{min}, T_{max}	0.900, 1.000	0.807, 1.000	0.573, 1.000	0.944, 0.971
No. measured reflections	7622	7854	2387	6763
No. independent reflections	1229	1253	1069	1468
No. observed [$I > 2\sigma(I)$] reflections	1191	1189	877	1020
R_{int}	0.036	0.029	0.029	0.054
$(\sin \theta/\lambda)_{max}$ (Å^{-1})	0.629	0.629	0.601	0.529
Refinement				
$R[F^2 > 2\sigma(F^2)]$, $wR(F^2)$, S	0.031, 0.085, 1.09	0.032, 0.085, 1.11	0.044, 0.135, 1.08	0.121, 0.425, 1.71
No. of reflections	1229	1253	1069	1468
No. of parameters	106	106	103	200
No. of restraints	1	1	-	-
$\Delta\rho_{max}$, $\Delta\rho_{min}$ (e Å^{-3})	0.17, -0.19	0.10, -0.17	0.19, -0.22	0.45, -0.41

Additional discussion of SCXRD data quality. A) Form I. The data for single crystals of Form I are well-behaved, although it is common to collect data on twinned crystals that initially appear hexagonal (*R*3) or pseudo-hexagonal ($P2_1$). Level C alerts are present in the SCXRD models for Form I primarily due to the use of R_{int} frame filters to remove frames or scans with $R_{int} > 0.5$ (or 50%). In most cases, the use of the frame filter was associated with an increase in the data collection target redundancy. **B) Form II.** SCXRD data quality for Form II varies by crystal growth method. Form II selectively crystallizes from the melt phase and crystal quality improves upon cycling heating/cooling around the melt temperature, 287 °C. Level C alerts are present in the SCXRD model for Form II due to use of R_{int} frame filters to remove frames or scans with $R_{int} > 0.5$ (or 50%) and for the significant difference in thermal ellipsoid size and anisotropicity between the central nitrogen atom and the nearest crystallographic neighbors. **C) Form III.** The data quality for crystals of Form III is generally poor due to crystal size (in particular, crystal thickness). Crystals grow in a thin flaky habit that leads to the observation of arcing along β that we attribute to twinning, high mosaicity, and/or stacking faults. Due to the size of Form III single crystals, the resolution is typically limited to 0.9 Å.

SI3. Variable-Temperature Crystallographic Parameters for Azangulene Solid Forms

Table SI2. Temperature-dependent lattice parameters, bowl depths, and anisotropic displacement parameters, W^{11}, measured from azangulene Forms I, II, and III.

T (K)	*a*-axis (Å)	*b*-axis (Å)	*c*-axis (Å)	*V* (Å^3)	ρ (g/cm^3)	R_1/wR_2 (%/%)	Flack	Bowl Depth (Å)	*N* W^{11}	C_1 W^{11}
**Form I** – Sample 1										
100	17.4358(3)	10.0783(2)	6.81961(13)	1198.36(4)	1.592	2.74/7.90	0.01	0.861	0.0252	0.0238
110	17.4372(4)	10.0808(2)	6.8254(2)	1199.78(5)	1.590	3.35/8.69	0.2	0.860	0.0264	0.0251
120	17.4461(3)	10.0827(3)	6.82870(10)	1201.19(4)	1.586	3.13/8.48	0.04	0.863	0.0272	0.0266
130	17.4445(3)	10.0816(2)	6.83353(14)	1201.80(4)	1.582	2.91/8.00	0.1	0.859	0.0279	0.0280
140	17.4460(3)	10.0834(2)	6.84010(10)	1203.28(4)	1.575	2.99/8.40	0.1	0.859	0.0283	0.0290
150	17.4561(3)	10.0871(2)	6.84290(10)	1204.91(4)	1.566	3.12/8.49	0.05	0.860	0.0308	0.0285
160	17.4608(3)	10.0867(2)	6.84940(10)	1206.33(4)	1.556	2.94/7.81	-0.07	0.860	0.0305	0.0291
170	17.4630(3)	10.0901(2)	6.85370(10)	1207.65(4)	1.544	2.82/7.91	0.2	0.860	0.0314	0.0318
180	17.4639(3)	10.0940(2)	6.86010(10)	1209.30(4)	1.530	3.07/8.50	-0.07	0.860	0.0340	0.0327
190	17.4748(3)	10.0939(2)	6.86450(10)	1210.82(4)	1.514	2.94/7.66	-0.12	0.858	0.0343	0.0336
200	17.4779(3)	10.0977(2)	6.86930(10)	1212.34(4)	1.497	2.93/7.89	-0.11	0.858	0.0359	0.0353
225	17.4911(3)	10.1042(2)	6.88420(10)	1216.67(4)	1.474	2.99/7.91	-0.01	0.857	0.0388	0.0383
250	17.4960(3)	10.1097(2)	6.89900(10)	1220.29(4)	1.448	3.04/8.19	0.06	0.853	0.0434	0.0411
275	17.5142(3)	10.1181(3)	6.91510(10)	1225.43(5)	1.416	3.06/8.38	-0.12	0.855	0.0466	0.0454
300	17.5297(3)	10.1232(3)	6.92640(10)	1229.14(5)	1.380	3.22/8.51	-0.12	0.850	0.0519	0.0490
**Form I** – Sample 2										
325	17.5311(12)	10.1226(7)	6.9250(4)	1228.91(14)	1.346	6.99/20.07	-0.4	0.887		
350	17.5533(10)	10.1338(7)	6.9388(4)	1234.29(13)	1.307	7.09/23.18	-0.1	0.883		
375	17.5638(9)	10.1367(6)	6.9545(4)	1238.17(12)	1.265	7.86/26.57	-0.3	0.867		
393	17.5764(12)	10.1470(9)	6.9642(3)	1242.05(15)	1.220	14.73/46.90	-0.1	0.853		
**Form II** – Sample 1										
120	7.3003(6)	10.3636(17)	15.7540(14)	1187.6(2)	1.606	6.69/19.97	-	0.000	0.1220	0.0496
150	7.3268(3)	10.3596(4)	15.7620(6)	1192.18(8)	1.600	4.44/13.49	-	0.000	0.1441	0.0529
200	7.3732(3)	10.3776(4)	15.7626(6)	1201.94(8)	1.580	5.58/17.86	-	0.000	0.1674	0.0635
225	7.3979(3)	10.3741(4)	15.7669(5)	1205.90(7)	1.556	4.64/14.46	-	0.000	0.1788	0.0685
250	7.4272(3)	10.3774(4)	15.7696(6)	1211.25(9)	1.526	4.94/14.63	-	0.000	0.2100	0.0823
275	7.4514(4)	10.3894(5)	15.7669(8)	1216.48(10)	1.489	5.29/16.32	-	0.000	0.1220	0.0496
300	7.4811(4)	10.3943(5)	15.7552(8)	1220.89(11)	1.448	4.77/15.78	-	0.000	0.1441	0.0529
325	7.5096(5)	10.4006(6)	15.7545(8)	1226.14(12)	1.403	5.72/17.89	-	0.000		
350	7.5441(4)	10.4019(6)	15.7435(8)	1231.00(12)	1.353	6.40/21.33	-	0.000		
375	7.5750(6)	10.4198(8)	15.7350(15)	1237.45(18)	1.299	6.17/21.06	-	0.000		
395	7.6102(7)	10.4168(8)	15.7271(12)	1242.12(18)	1.242	5.25/17.07	-	0.000		
**Form III**										
110	18.600(5)	3.7710(7)	18.913(4)	1197.6(5)	1.594	13.61/42.93	-	0.328	0.1348	0.0670
140	18.601(4)	3.7766(6)	18.932(3)	1201.1(4)	1.588	13.01/44.64	-	0.295	0.1329	0.0790
160	18.585(3)	3.7892(6)	18.943(3)	1205.3(4)	1.583	12.08/42.53	-	0.255	0.1847	0.0777
200	18.594(3)	3.8044(5)	18.951(4)	1211.4(4)	1.561	13.47/41.90	-	0.185	0.2195	0.0812
290	18.624(5)	3.8450(9)	19.032(5)	1232.4(6)	1.515	11.14/40.44	-	0.097	0.2327	0.1038

Table SI3. Coefficient of linear thermal expansion (CTLE) for azangulene Forms I and II measured above room temperature along the three crystallographic axes.

	Form I			Form II		
	a-axis (ΔÅ/Å)	*b*-axis (ΔÅ/Å)	*c*-axis (ΔÅ/Å)	*a*-axis (ΔÅ/Å)	*b*-axis (ΔÅ/Å)	*c*-axis (ΔÅ/Å)
300 K	0.00000	0.00000	0.00000	0.00000	0.00000	0.00000
325 K	0.00007	-0.00006	-0.00020	0.00381	0.00061	-0.00003
350 K	0.00135	0.00105	0.00179	0.00842	0.00073	-0.00074
375 K	0.00195	0.00133	0.00406	0.01255	0.00245	-0.00128
393/5 K	0.00266	0.00235	0.00546	0.01726	0.00216	-0.00178
CLTE (MK^{-1})	28.646	25.280	58.682	181.651	22.786	-18.774

Table SI4. Temperature-dependent anisotropic displacement parameters and ratio of anisotropic displacement parameters measured from the three azangulene solid forms, Form I, Form II, and Form III.

T (K)	W_N^{11}	W_C^{11}	W_O^{11}	W_N^{11} / W_C^{11}	W_N^{11} / W_O^{11}	W_N^{33} / W_N^{11}
**Form I**						
100	0.0252	0.0238	0.0356	1.057	0.708	0.596
110	0.0264	0.0251	0.0384	1.049	0.686	0.605
120	0.0272	0.0266	0.0389	1.023	0.699	0.679
130	0.0279	0.0280	0.0407	0.996	0.684	0.730
140	0.0283	0.0290	0.0422	0.976	0.671	0.738
150	0.0308	0.0285	0.0448	1.078	0.687	0.662
160	0.0305	0.0291	0.0460	1.050	0.664	0.697
170	0.0314	0.0318	0.0475	0.988	0.661	0.714
180	0.0340	0.0327	0.0506	1.040	0.671	0.654
190	0.0343	0.0336	0.0520	1.021	0.659	0.708
200	0.0359	0.0353	0.0546	1.017	0.658	0.701
225	0.0388	0.0383	0.0604	1.013	0.642	0.718
250	0.0434	0.0411	0.0669	1.058	0.649	0.692
275	0.0466	0.0454	0.0724	1.027	0.643	0.720
300	0.0519	0.0490	0.0784	1.058	0.662	0.697
**Form II**						
120	0.1220	0.0496	0.0802	2.460	1.520	0.115
150	0.1441	0.0529	0.0894	2.724	1.613	0.128
200	0.1674	0.0635	0.1043	2.639	1.605	0.138
225	0.1788	0.0685	0.1138	2.612	1.571	0.130
300	0.2100	0.0823	0.1388	2.551	1.514	0.160
**Form III**						
110	0.1348	0.0670	0.0875	2.010	1.540	0.130
140	0.1329	0.0790	0.1847	1.682	0.719	0.329
160	0.1847	0.0777	0.0797	2.376	2.317	0.106
200	0.2195	0.0812	0.0967	2.704	2.270	0.078
290	0.2327	0.1038	0.1260	2.242	1.847	0.143

The W_N^{11} quantifies the length of the long axis of the crystallographic anisotropic displacement parameter for the nitrogen atom. The W_C^{11} quantifies the length of the long axis of the crystallographic anisotropic displacement parameter for the carbon atom adjacent to the nitrogen atom in azangulene. The W_O^{11} quantifies the length of the long axis of the crystallographic anisotropic displacement parameter for the oxygen atom. The ratios of W_N^{11} / W_C^{11} and W_N^{11} / W_O^{11} quantify the degree to which the nitrogen atom W_N^{11} is elongated relative to the whole molecule. A value less than or near 1 ($0.9 < W_N^{11} / W_X^{11} < 1.1$) for W_N^{11} / W_C^{11} and W_N^{11} / W_O^{11} indicates that the elongation of the nitrogen atom W_N^{11} is not unique to the nitrogen atom but is a property of the whole molecule. A value greater than 1 for W_N^{11} / W_C^{11} and W_N^{11} / W_O^{11} indicates that the nitrogen atom is significantly elongated relative to the whole molecule. The W_N^{33} / W_N^{11} is a ratio that quantifies the anisotropicity of the nitrogen atom thermal ellipsoid elongation. A value close to 1 indicates that the nitrogen atom thermal ellipsoid is spherical, while a value close to 0 indicates that the nitrogen atom thermal ellipsoid is highly anisotropic.

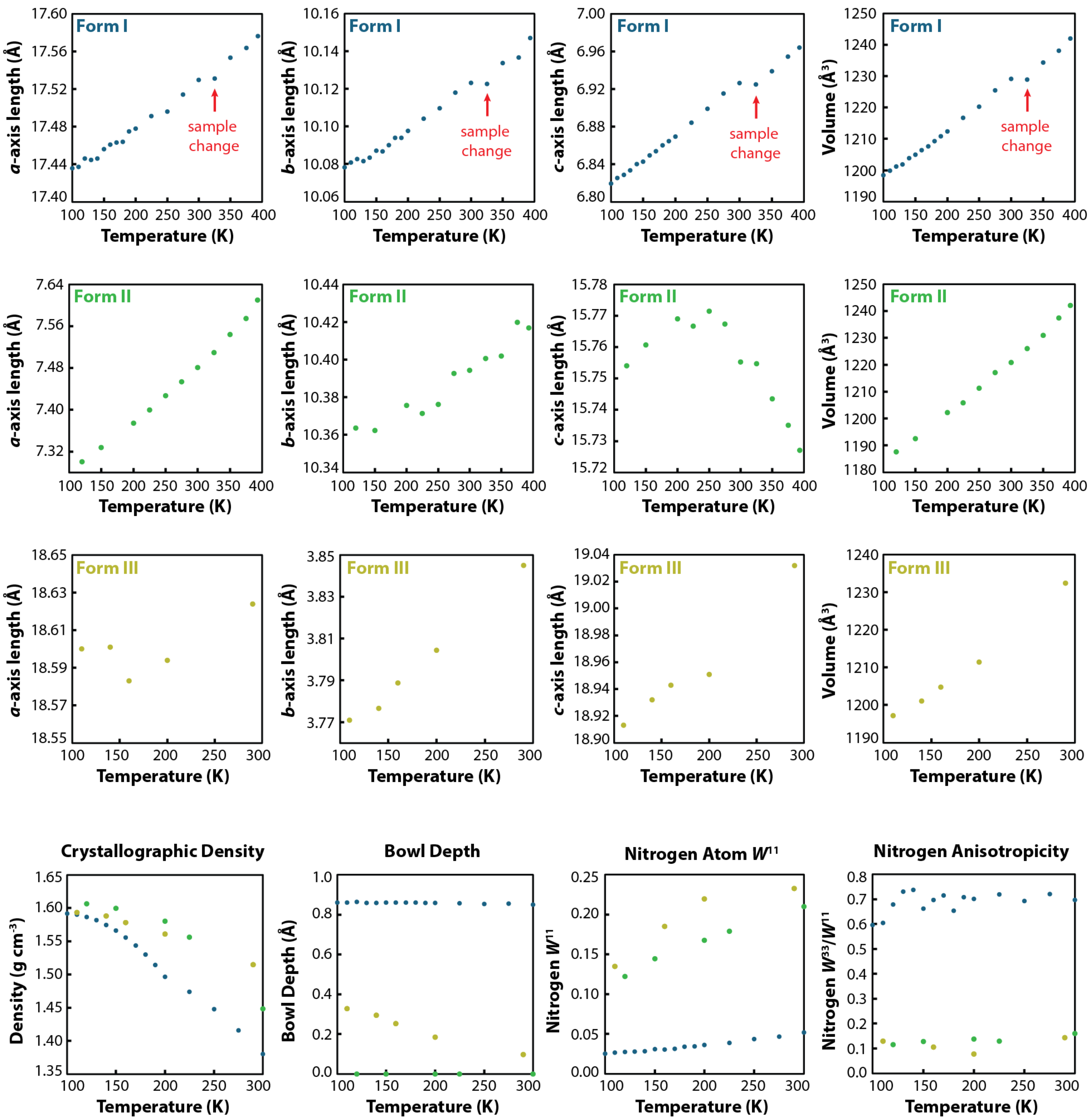

Figure SI2. Variable-temperature unit cell parameters for Form I (blue, top row), Form II (green, second row from top), and Form III (gold, second row from bottom) as well as the change in crystallographic density, bowl depth, and nitrogen atom W^{11}, and nitrogen atom anisotropicity for all three solid forms of azangulene (bottom row) in which data points for Form I are shown in blue, Form II are shown in green, and Form III are shown in gold. For the nitrogen atom anisotropicity ratio, a value close to 1 indicates that the nitrogen atom thermal ellipsoid is spherical, while a value close to 0 indicates that the nitrogen atom thermal ellipsoid is highly anisotropic.

SI4. van't Hoff Analysis of Form I and Form III Crystallographic Bowl Depths

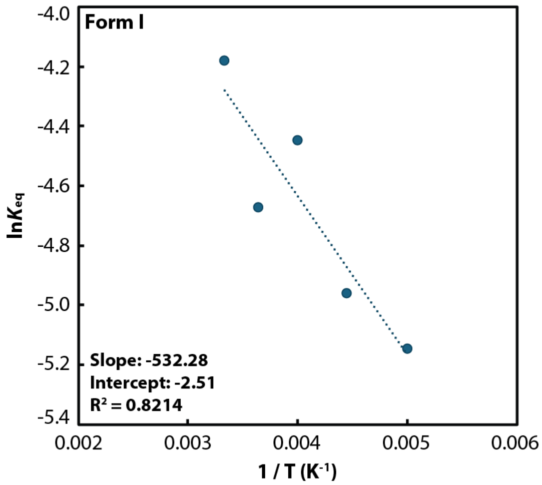

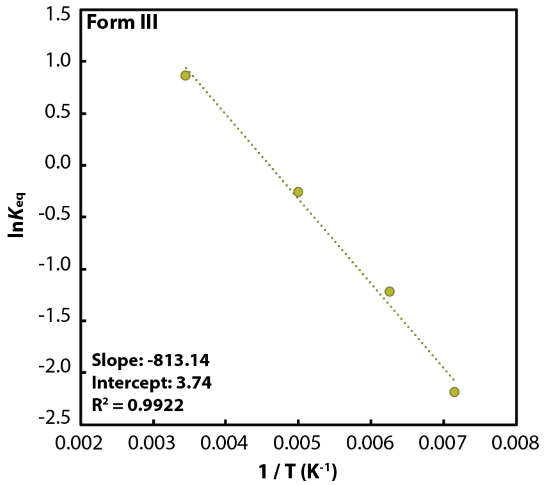

Figure SI3. van't Hoff plots for Form I (left) and for Form III (right) based on the data presented in Table SI5. The slopes, intercepts, and R^2 values are given in the corresponding plot from which the changes in enthalpy and entropy were determined.

Table SI5. Solid form and temperature-dependent bowl depths for azangulene used for a two-state van't Hoff analysis to estimate the change in enthalpy and entropy associated with planarization of azangulene in the solid state.

Temperature (K)	Bowl Depth (Å)	Bowl Population	Flat Population	K_{eq} [Flat]/[Bowl]	lnK_{eq}	$\Delta G°$ (kcal / mol)
**Form I**						
120	0.863	1.000	0.000	0.000		
200	0.858	0.994	0.006	5.83 x 10^{-3}	-5.145	2.044
225	0.857	0.993	0.007	7.00 x 10^{-3}	-4.962	2.218
250	0.853	0.988	0.012	11.72 x 10^{-3}	-4.446	2.209
275	0.855	0.991	0.009	9.36 x 10^{-3}	-4.672	2.553
300	0.850	0.985	0.015	15.29 x 10^{-3}	-4.180	2.492
			**$\Delta H°$ = 1.058 kcal / mol**		**$\Delta S°$ = -4.984 cal / mol** (R^2 = 0.8214)	
**Form III**						
110	0.328	1.000	0.000	0.000		
140	0.295	0.899	0.101	0.112	-2.190	0.609
160	0.255	0.771	0.229	0.296	-1.216	0.387
200	0.185	0.564	0.436	0.773	-0.258	0.102
290	0.097	0.296	0.704	2.381	-0.868	-0.500
			**$\Delta H°$ = 1.616 kcal / mol**		**$\Delta S°$ = 7.434 cal / mol** (R^2 = 0.9922)	

SI5. Polarization Hysteresis Measurement

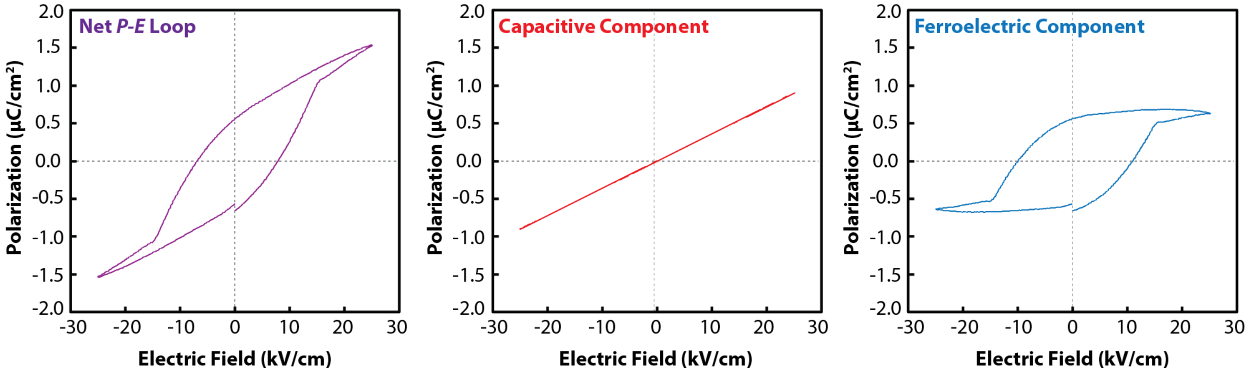

Figure SI4. Net polarization hysteresis (*P*-*E*) loop (purple, left) measured from a single crystal of Form I azangulene according to the protocol in Section SI 1.14. Data were collected at 38 °C and under ambient atmosphere. The capacitive component of the polarization hysteresis loop was extracted during post-processing and a plot of the capacitive component as a function of applied electric field strength is given (red, center). The capacitive component was removed from the net *P*-*E* loop to yield the ferroelectric component from which the saturation polarization (P_S), the remnant polarization (P_r), and coercive field strength (E_C) were determined.

SI6. Differential Scanning Calorimetry

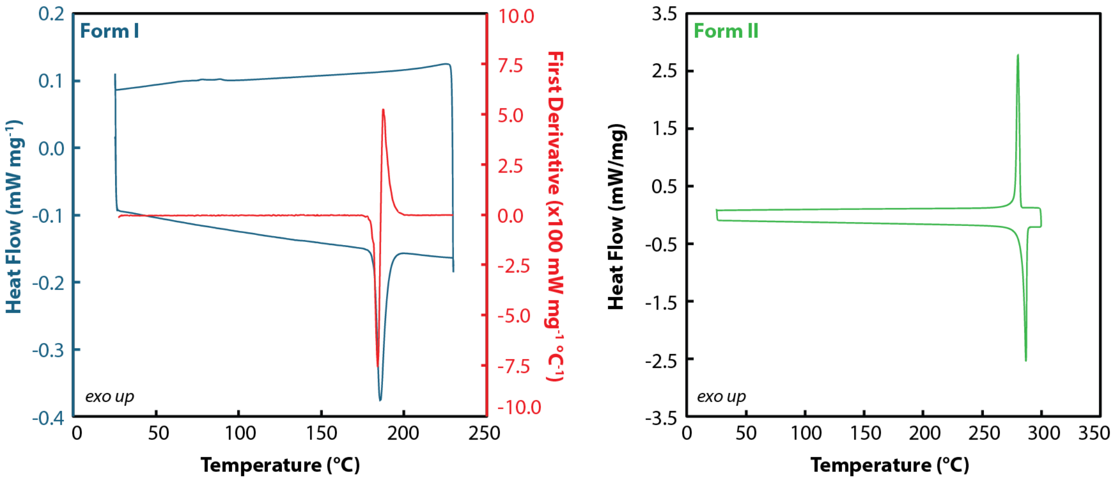

Figure SI5. Differential scanning calorimetry (DSC) traces for Form I (blue, left) and Form II (green, right). The DSC trace of Form I shows an irreversible phase transition at 185 °C (upon heating), which is the phase transition between Form I and Form II. The phase transition temperature between Form I and Form II was determined from the first derivative of the DSC trace of Form I (red). Form II shows a reversible phase transition at 287 °C (upon heating), which is a melt event.

SI7. Slurry Equilibration of Azangulene Solid Forms

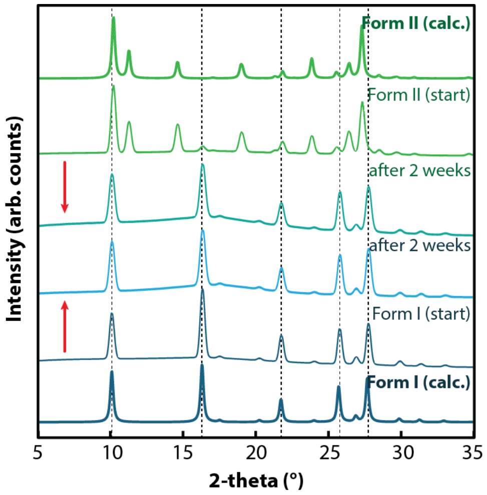

Figure SI6. Powder X-ray diffraction (PXRD) patterns of Form I and Form II azangulene before and after two weeks of slurry equilibration in benzene. The bolded PXRD patterns at the top and bottom of the stacked diffractograms were calculated from the single-crystal X-ray diffraction (SCXRD) models of the corresponding azangulene polymorph, highlighting the agreement in peak position and relative peak intensities between the samples of Form I or Form II and the SCXRD model at the start of the slurry equilibration. The PXRD patterns that result from the conversion of both slurry experiments are indicated by the red arrows; after two weeks of equilibration in benzene at room temperature, Form I stays Form I and Form II converts into Form I.

SI8. Computational Investigation of Gas-Phase Azangulene Whole-Molecule Inversion

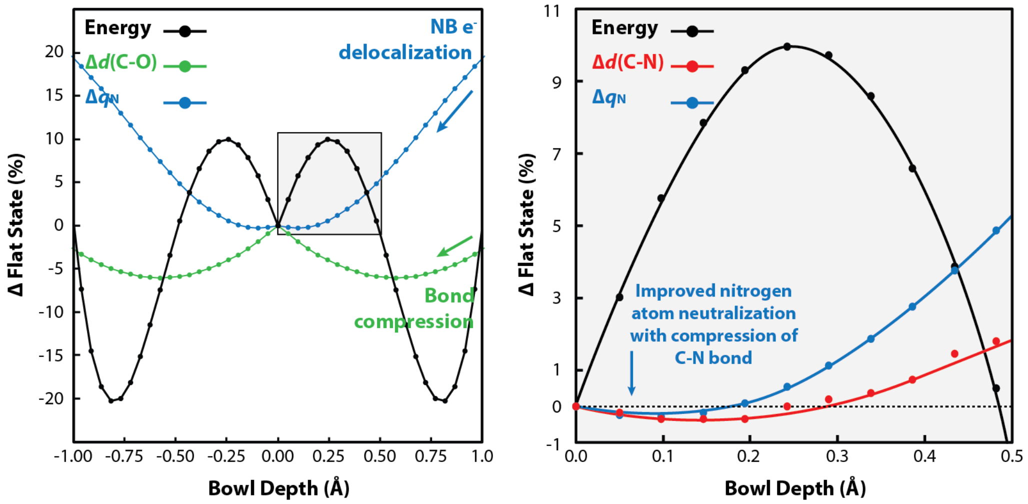

Figure SI7. Plots of computed azangulene molecular properties as a function of bowl depth. The left plot shows the conformational inversion enthalpy landscape as well as two key molecular properties that were extracted from the corresponding energy calculations: the partial charge partitioned to the central nitrogen atom (q_N, blue) using a natural atomic orbital method and the compression of the bond length between the bridging oxygen atom and the adjacent carbon atom, *d*(C-O) (green). The right plot shows the correlation between q_N and the bond length between the central nitrogen atom and the adjacent carbon atom, *d*(C-N) (red). Compression of *d*(C-N) is correlated with neutralization of q_N beyond what is achieved in the flat conformer.

Table SI6. Energies and molecular characteristics of azangulene geometries used to compute the conformation enthalpy surface for whole-molecule inversion. The characteristics for each geometry are organized by the bowl depth of the corresponding geometry. The computed values are given for optimized energy (relative to the optimized conformational geometry), partial charge assigned to the central nitrogen atom (q_N) using natural atomic orbitals, and the bond distance between the bridging oxygen atom and the adjacent carbon atom, *d*(C-O). For the plot in Figure SI13, the molecular properties were compared to the corresponding property of the flat geometry and scaled for ease of interpretation using the following equation, where "*Y*" is the property, Y_X is the property of the geometry of interest, Y_{flat} is the property of the flat geometry, and γ is the scaling factor:

$$Y_{plot} = \gamma(Y_X - Y_{flat}) / Y_{flat} \times 100\%$$

Bowl Depth (Å)	Rel. Energy (kcal mol^{-1})	Rel. Energy (% Flat)	q_N (NAO)	Δq_N (% Flat)	*d*(C-O) (Å)	Δd(C-O) (% Flat)
0.000	0.6178	0.000	-0.3990	0.000	1.3880	0.000
0.050	0.7108	3.011	-0.3988	-0.238	1.3875	-0.973
0.098	0.7955	5.751	-0.3988	-0.301	1.3870	-1.855
0.146	0.8600	7.839	-0.3989	-0.188	1.3866	-2.648
0.194	0.9050	9.294	-0.3991	0.088	1.3862	-3.368
0.242	0.9254	9.956	-0.3994	0.526	1.3858	-3.999
0.290	0.9180	9.718	-0.3999	1.128	1.3855	-4.539
0.338	0.8827	8.576	-0.4005	1.867	1.3852	-5.007
0.386	0.8210	6.578	-0.4012	2.757	1.3850	-5.367
0.434	0.7371	3.863	-0.4020	3.759	1.3849	-5.674
0.482	0.6330	0.489	-0.4029	4.575	1.3848	-5.872
0.530	0.5135	-3.378	-0.4039	6.090	1.3847	-5.998
0.578	0.3877	-7.450	-0.4049	7.381	1.3847	-6.034
0.626	0.2631	-11.484	-0.4060	8.734	1.3847	-5.980
0.674	0.1492	-15.170	-0.4071	10.150	1.3848	-5.854
0.722	0.0576	-18.134	-0.4083	11.579	1.3849	-5.638
0.770	0.0000	-20.000	-0.4094	13.007	1.3851	-5.331
0.818	-0.0096	-20.309	-0.4105	14.436	1.3853	-4.953
0.866	0.0418	-18.646	-0.4116	15.827	1.3855	-4.485
0.915	0.1692	-14.523	-0.4127	17.155	1.3858	-3.927
0.963	0.3909	-7.347	-0.4137	18.421	1.3862	-3.296
1.011	0.7207	3.332	-0.4146	19.586	1.3866	-2.576

SI9. Computational Investigation of Gas-Phase Azangulene Nitrogen Atom Inversion Enthalpy Surface

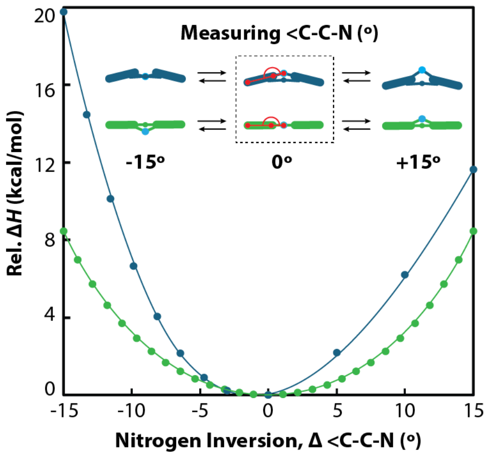

Figure SI8. Enthalpy surface for nitrogen atom inversion for azangulene that is conformationally locked into a bowl geometry (blue) or a flat geometry (flat). The degree of nitrogen atom inversion is quantified by the angle between the central nitrogen atom, the carbon atom (C_1) directly bound to the central nitrogen atom, and the carbon atom *para* to C_1. This angle is referred to as the <C-C-N angle, in which an angle of 180° is defined as 0° of nitrogen inversion. The nitrogen atom was oscillated +/-15° from this angle to yield the enthalpy surface shown in this figure.

Table SI7. Table of optimized energies calculated for nitrogen atom inversion of azangulene that is conformationally locked into a flat geometry or a bowl geometry. The degree of nitrogen atom inversion is quantified by the angle between the central nitrogen atom, the carbon atom (C_1) directly bound to the central nitrogen atom, and the carbon atom *para* to C_1 (this is illustrated in the inset for Figure SI14). This angle is referred to as the <C-C-N angle, in which an angle of 180° is defined as 0° of nitrogen inversion. Because conformationally flat azangulene has an internal plan of mirror symmetry, the energy values for oscillation of the nitrogen atom to negative angle positions were generated by symmetry.

Flat Azangulene

Δ <C-C-N (°)	ΔE_{opt} (kcal/mol)
0.00	0.00
1.07	0.03
2.14	0.13
3.21	0.30
4.29	0.53
5.36	0.83
6.43	1.22
7.50	1.68
8.57	2.25
9.64	2.92
10.71	3.71
11.79	4.64
12.86	5.73
13.93	6.99
15.00	8.45

Bowl Azangulene

Δ <C-C-N (°)	ΔE_{opt} (kcal/mol)
15.00	11.63
10.00	6.20
5.00	2.20
0.00	0.00
-3.00	0.24
-4.71	0.91
-6.43	2.15
-8.14	4.04
-9.86	6.67
-11.57	10.13
-13.29	14.48
-15.00	19.78

SI10. Nucleus-Independent Chemical Shift (NICS) Ring Current Maps

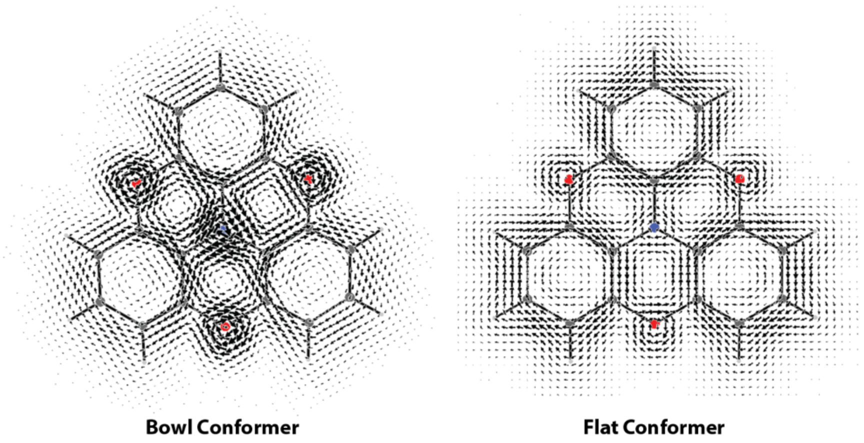

Figure SI9. Nucleus-independent chemical shift (NICS) ring current density maps calculated using SYSMOIC[14] from energy calculation outputs generated by Gaussian[11] *via* the WebMO[12] GUI (B3LYP/6-31(d)). Current density maps were probed 1.25 Å above the molecule's surface. Clockwise indicates diatropic ring current and counterclockwise indicates paratropic ring current.

SI11. Computational Library of Azangulene Derivatives

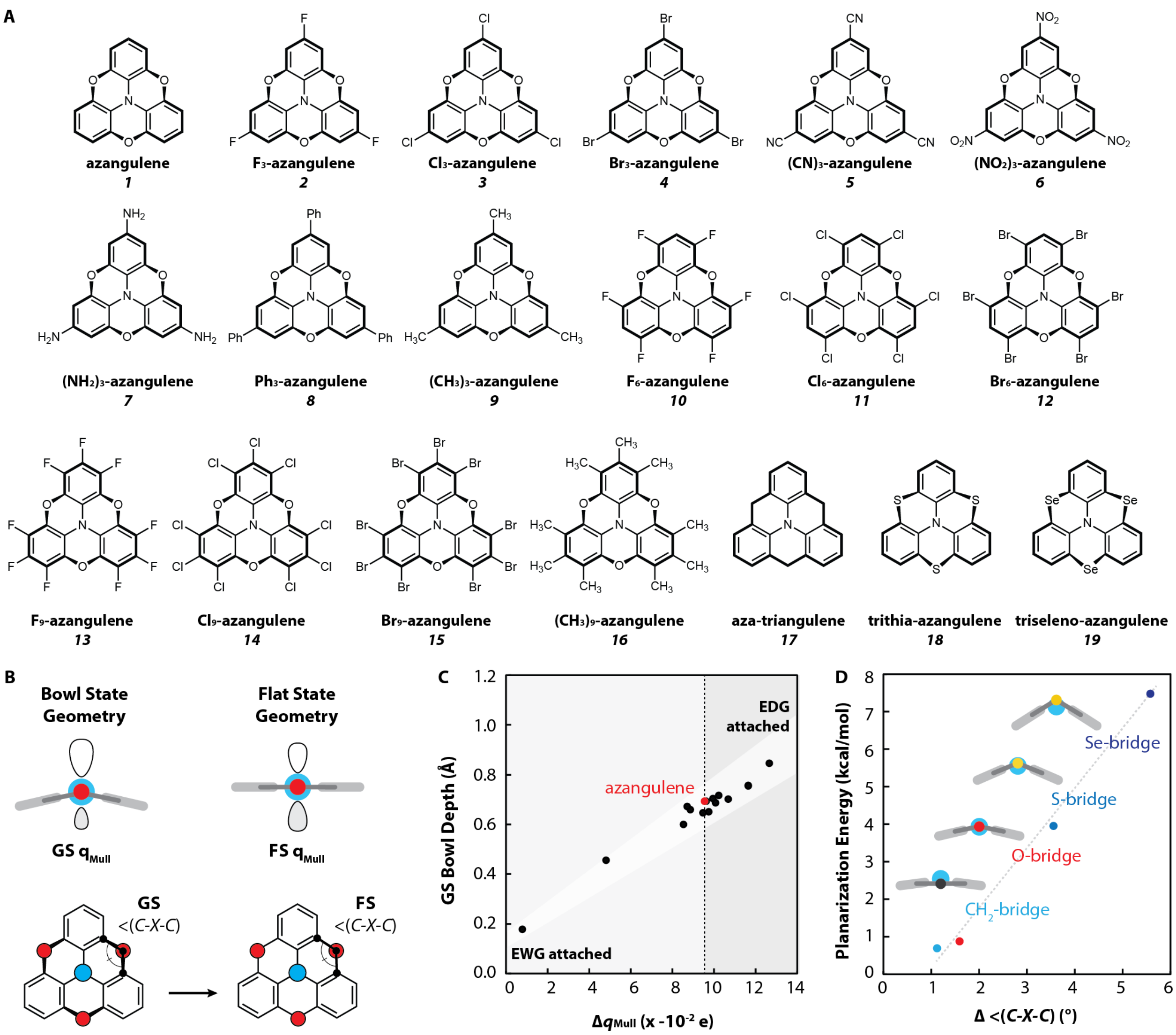

Figure SI10. A) Molecular structures, compound names, and compound numbers for compounds analyzed in the computational library; **B)** profile and top-down views of azangulene in the bowl state (GS) and flat state (FS), highlighting how the bridging angles, <(*C*-*X*-*C*), and Mulliken populations, q_{Mull}, are defined; **C)** the change in partial charge of the central N atom (Δq_{Mull}) plotted against GS bowl depth; **D)** the change in bridging atom angle between the GS and FS, Δ<(*C*-*X*-*C*), plotted against the difference in computed energy.

Table SI8. Computed molecular energies and properties for the equilibrium bowl conformer (GS) and the flat conformer (FS) of each azangulene derivative or analogue. The molecular structures of each compound are given in Figure SI15. The difference in energy between the bowl and flat geometries for each derivative is given by ΔE in kcal/mol. The partial charge partitioned to the central nitrogen atom in the bowl and flat conformers are given as **GS** q_{Mull} and **FS** q_{Mull}, respectively. The bridging atom angle for the bowl and flat conformers are given as **GS** <(*C*-*X*-*C*) and **FS** <(*C*-*X*-*C*), respectively, for the series of azangulene analogues with substitutions of the bridging atoms. Diagrams illustrating measurement of q_{Mull} and <(*C*-*X*-*C*) are given in Figure SI15.

Compound	Group	**GS** Bowl Depth (Å)	ΔE (kcal/mol)	**GS** q_{Mull}	**FS** q_{Mull}	Δq_{Mull}	**GS** <(*C*-*X*-*C*)	**FS** <(*C*-*X*-*C*)
1	$-H_3$	0.693	0.693	-0.687395	-0.782871	0.095476	117.45	118.57
2	$-F_3$	0.756	1.090	-0.675149	-0.791564	0.116415		
3	$-Cl_3$	0.717	0.780	-0.685576	-0.787698	0.102122		
4	$-Br_3$	0.704	0.723	-0.689681	-0.789085	0.099404		
5	$-(CN)_3$	0.456	0.109	-0.744961	-0.792892	0.047931		
6	$-(NO_2)_3$	0.178	-0.001	-0.789163	-0.797085	0.007922		
7	$-(NH_2)_3$	0.847	5.952	-0.655865	-0.78243	0.126565		
8	$-Ph_3$	0.673	0.506	-0.698041	-0.784971	0.08693		
9	$-(CH_3)_3$	0.693	1.642	-0.691886	-0.787902	0.096016		
10	$-F_6$	0.659	0.520	-0.72586	-0.814504	0.088644		
11	$-Cl_6$	0.601	0.407	-0.725841	-0.810987	0.085146		
12	$-Br_6$	0.646	0.480	-0.722023	-0.816639	0.094616		
13	$-F_9$	0.754	0.942	-0.709045	-0.825436	0.116391		
14	$-Cl_9$	0.651	0.550	-0.717356	-0.814719	0.097363		
15	$-Br_9$	0.701	0.646	-0.715128	-0.821754	0.106626		
16	$-(CH_3)_9$	0.688	2.322	-0.70847	-0.809057	0.100587		
17	$-CH_2-$	0.666	0.878	-0.806827	-0.876223	0.069396	112.24	113.82
18	-S-	0.841	3.960	-0.725516	-0.794794	0.069278	97.82	101.37
19	-Se-	1.081	7.481	-0.71756	-0.814349	0.096789	91.44	97.02

SI12. ORTEP for Determined X-Ray Crystal Structures

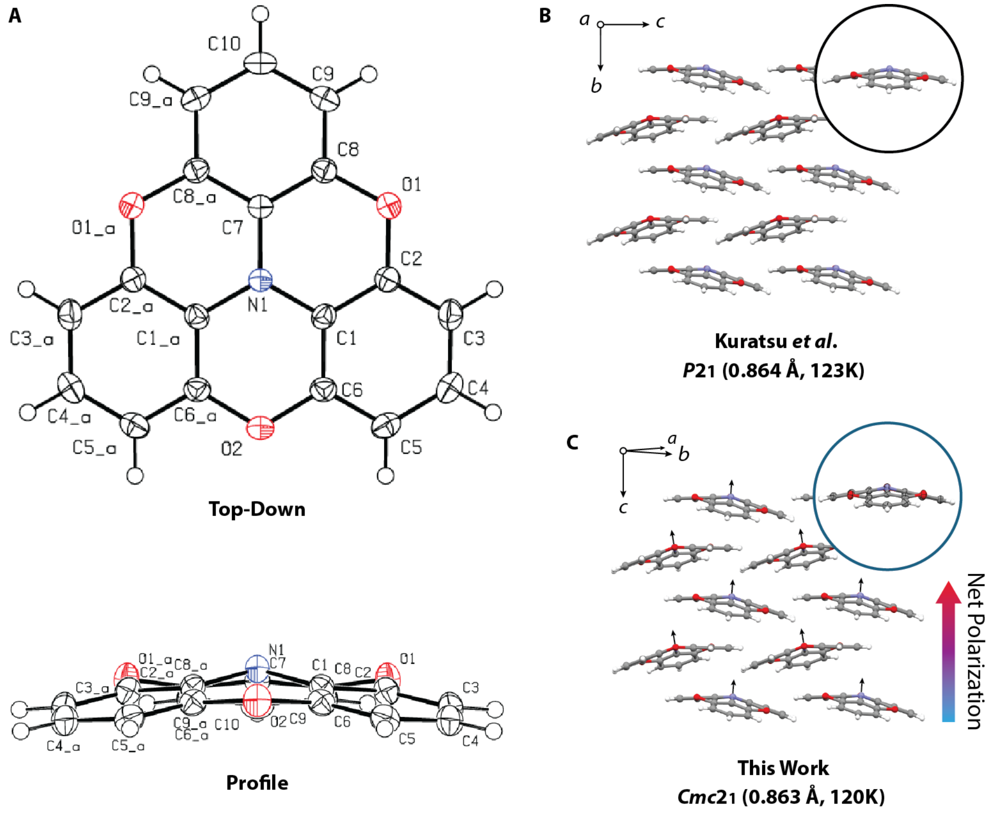

Figure SI10. A) Structure of azangulene (Form I at 120K) with displacement ellipsoids drawn at the 50% probability level and the labeling scheme provided for all unique non-hydrogen atoms; **B)** crystal packing motif solved in $P2_1$ and single-molecule conformation of azangulene previously published by Kuratsu *et al*.[1] (CCDC 262144), reproduced from the main text for clarity; **C)** crystal packing motif of azangulene solved in $Cmc2_1$ and single-molecule conformation collected at 120 K, reproduced from the main text for clarity.

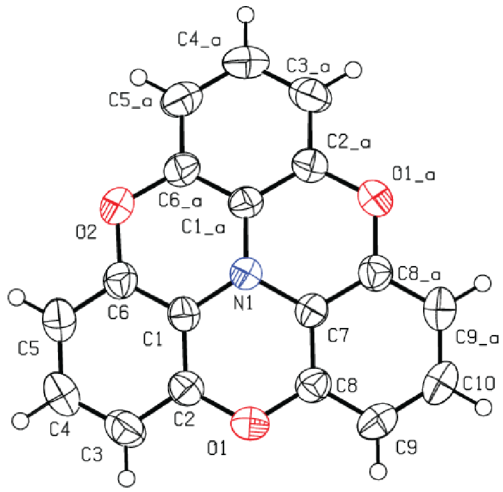

Top-Down

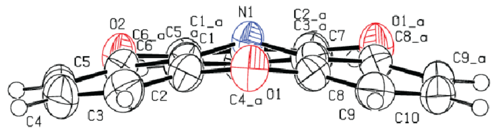

Profile

Figure SI11. Structure of azangulene (Form I at 300K) with displacement ellipsoids drawn at the 50% probability level and the labeling scheme provided for all unique non-hydrogen atoms.

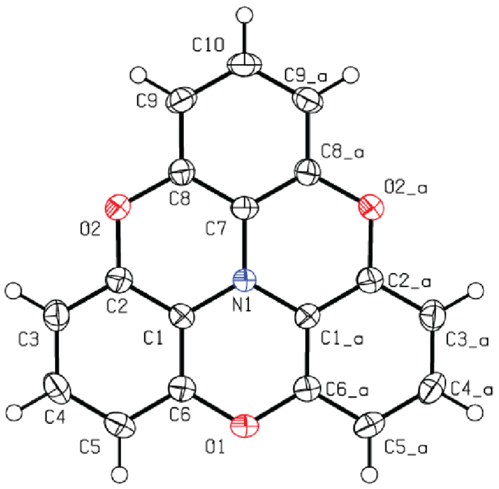

Top-Down

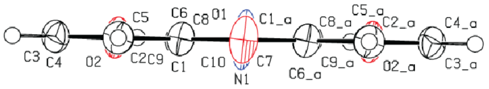

Profile

Figure SI12. Structure of azangulene (Form II at 150K) with displacement ellipsoids drawn at the 50% probability level and the labeling scheme provided for all unique non-hydrogen atoms.

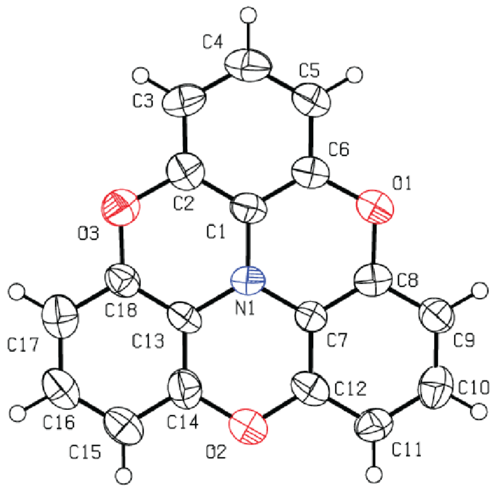

Top-Down

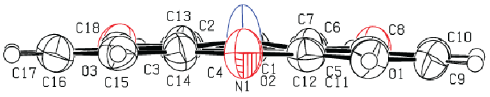

Profile

Figure SI13. Structure of azangulene (Form III at 160K) with displacement ellipsoids drawn at the 50% probability level and the labeling scheme provided for all unique non-hydrogen atoms.

SI13. References

[1]Kuratsu, M.; Kozaki, M.; Okada, K. *Angew. Chem. Int. Ed.* **2005**, *44* (26), 4056-4058.

[2]Breimaier, S.; Winter, R. F. *Eur. J. Org. Chem.* **2021**, 4690-4670.

[3]Oxford Diffraction, CrysAlisPRO Agilent Technologies UK Ltd, Yarnton, England.

[4]Dolomanov, O. V.; Bourhis, L. J.; Gildea, R. J.; Howard, J. A.; Puschman, H. *J. Appl. Crystallogr.* **2009**, *42*, 339-341.

[5]Sheldrick, G. M. *Acta Cryst. A* **2015**, *71*, 3-8.

[6]Sheldrick, G. M. *Acta Cryst. C* **2015**, *71*, 3-8.

[7]Macrae, C. F.; Sovago, I.; Cottrell, S. J.; Galek, P. T. A.; McCabe, P.; Pidcock, E.; Platings, G. P.; Shields, G. P.; Stevens, J. S.; Towler, M.; Wood, P. A. *Acta Cryst. A* **2020**, *53*, 226-235.

[8]Zhou, L.; Liu, Q. *J. Phys. Chem. B* **2014**, *118*, 4069-4079.

[9]Grosse-Kunstleve, R. W.; Adams, P. D. *J. Appl. Cryst.* **2002**, *35*, 477-480.

[10]Frisch, M. J.; Trucks, G. W.; Schlegel, H. B.; Scuseria, G. E.; Robb, M. A.; Cheeseman, J. R.; Scalmani, G.; Barone, V.; Petersson, G. A.; Nakatsuji, H.; Li, X.; Caricato, M.; Marenich, A. V.; Bloino, J.; Janesko, B. G.; Gomperts, R.; Mennucci, B.; Hratchian, H. P.; Ortiz, J. V.; Izmaylov, A. F.; Sonnenberg, J. L.; Williams-Young, D.; Ding, F.; Lipparini, F.; Egidi, F.; Goings, J.; Peng, B.; Petrone, A.; Henderson, T.; Ranasinghe, D.; Zakrzewski, V. G.; Gao, J.; Rega, N.; Zheng, G.; Liang, W.; Hada, M.; Ehara, M.; Toyota, K.; Fukuda, R.; Hasegawa, J.; Ishida, M.; Nakajima, T.; Honda, Y.; Kitao, O.; Nakai, H.; Vreven, T.; Throssell, K.; Montgomery Jr., J. A.; Peralta, J. E.; Ogliaro, F.; Bearpark, M. J.; Heyd, J. J.; Brothers, E. N.; Kudin, K. N.; Staroverov, V. N.; Keith, T. A.; Kobayashi, R.; Normand, J.; Raghavachari, K.; Rendell, A. P.; Burant, J. C.; Iyengar, S. S.; Tomasi, J.; Cossi, M.; Millam, J. M.; Klene, M.; Adamo, C.; Cammi, R.; Ochterski, J. W.; Martin, R. L.; Morokuma, K.; Farkas, O.; Foresman, J. B.; Fox, D. J.; Gaussian 16 *Rev. C.01*, **2016**.

[11]Polik, W. F.; Schmidt, J. R. *WIREs Comput. Mol. Sci.* **2021**, *e1554.*

[12]Shao, Y.; Molnar, L. F.; Jung, Y.; Kussmann, J.; Ochsenfeld, C.; Brown, S. T.; Gilbert, A. T. B.; Slipchenko, L. V.; Levchenko, S. V.; O'Neill, D. P.; Jr, R. A. D.; Lochan, R. C.; Wang, T.; Beran, G. J. O.; Besley, N. A.; Herbert, J. M.; Lin, C. Y.; Voorhis, T. V.; Chien, S. H.; Sodt, A.; Steele, R. P.; Rassolov, V. A.; Maslen, P. E.; Korambath, P. P.; Adamson, R. D.; Austin, B.; Baker, J.; Byrd, E. F. C.; Dachsel, H.; Doerksen, R. J.; Dreuw, A.; Dunietz, B. D.; Dutoi, A. D.; Furlani, T. R.; Gwaltney, S. R.; Heyden, A.; Hirata, S.; Hsu, C.-P.; Kedziora, G.; Khalliulin, R. Z.; Klunzinger, P.; Lee, A. M.; Lee, M. S.; Liang, W.; Lotan, I.; Nair, N.; Peters, B.; Proynov, E. I.; Pieniazek, P. A.; Rhee, Y. M.; Ritchie, J.; Rosta, E.; Sherrill, C. D.; Simmonett, A. C.; Subotnik, J. E.; Iii, H. L. W.; Zhang, W.; Bell, A. T.; Chakraborty, A. K.; Chipman, D. M.; Keil, F. J.; Warshel, A.; Hehre, W. J.; Iii, H. F. S.; Kong, J.; Krylov, A. I.; Gill, P. M. W.; Head-Gordon, M. Advances in Methods and Algorithms in a Modern Quantum Chemistry Program Package. *Phys. Chem. Chem. Phys.* **2006**, *8* (27), 3172–3191.

[13]Sim, G. A. *J. Chem. Soc., Chem. Commun.* **1987**, *14*, 1118-1120.

[14]Monaco, G.; Summa, F. F.; Zanasi, R. *J. Chem. Inf. Model.* **2021**, *61* (1), 270-283.
